# The response relevance of visual stimuli modulates the P3 component and the underlying sensorimotor network

**DOI:** 10.1038/s41598-020-60268-z

**Published:** 2020-03-02

**Authors:** Dariusz Asanowicz, Krzysztof Gociewicz, Marcin Koculak, Karolina Finc, Kamil Bonna, Axel Cleeremans, Marek Binder

**Affiliations:** 1 Institute of Psychology, Jagiellonian University, Kraków, Poland; 2 Centre for Modern Interdisciplinary Technologies, Nicolaus Copernicus University, Toruń, Poland; 3 Consciousness, Cognition, and Computation Group, Université Libre de Bruxelles, Brussels, Belgium

**Keywords:** Cognitive neuroscience, Psychology

## Abstract

The functional meaning and neural basis of the P3b component of ERPs are still under debate. One of the main issues is whether P3b reflects only stimulus-related processes (stimulus evaluation hypothesis) or response-related processes as well (stimulus-response or S-R link activation hypothesis). Here, we conducted an EEG experiment examining whether P3b may indeed reflect an S-R link activation, followed by an fMRI experiment in which we explored the brain areas and functional connectivity possibly constituting the neural basis of these sensorimotor links. In both experiments, two successive visual stimuli, S1 and S2, were presented with a 1 sec interval, and responses were defined either by S1 or S2, while participants responded only after S2 onset. The obtained EEG results suggest that P3b may be interpreted in terms of the S-R link activation account, although further studies are needed to disentangle P3-related activity from overlapping anticipatory activity. The obtained fMRI results showed that processing of the relevant S1 involved activation of a distributed postero-anterior sensorimotor network, and increased strength of functional connectivity within this network. This network may underlie activation of the S-R links, thus possibly also the P3b component, forming a bridging step between sensory encoding and response execution.

## Introduction

In the present study, we conducted two experiments: an EEG experiment aimed at examining the issue of whether P3b may reflect processes related to activation of stimulus-response (S-R) links or “event files”, followed by an fMRI experiment aimed at exploring what may constitute the neural basis of these sensorimotor links.

## EEG Experiment

### Introduction

Over four decades have passed since Squires, Squires, and Hillyard^1^ described the P3b component (that we will further refer to as P3) - a major centro-parietal part of the P300 complex in the human event-related EEG potential (ERP) with a maximal positive deflection at parietal midline (usually the Pz site) at about 300–700 ms after stimulus onset. Years of extensive studies have shown that P3 emerges whenever a task-related or behaviorally-relevant stimulus is perceived, regardless of stimulus and response modalities, and changes in P3 amplitude and latency are related to a wide range of higher-level cognitive processes^2–4^. Yet, the functional meaning of P3 as well as its neural underpinnings are still under debate.

Answers to the question of “what is the underlying process reflected by P3?” usually fall into one of two general views. The first view is that P3 reflects stimulus processing only, and is neither related to nor affected by processes of response selection and preparation^5,6^. Seminal for this approach, the stimulus evaluation account maintains that P3 is a signature of comprehensive evaluation of perceived events. This evaluation entails processes of allocation of perceptual and/or attentional resources to event encoding and categorization^7–9^, and it is often assumed here that P3 amplitude reflects the amount of these resources or cognitive capacity involved in the stimulus evaluation^10,11^. The second view is that P3 reflects some processes of stimulus-response (S-R) translation or integration, a bridging step between sensory encoding and response execution^12,13^. Following this general idea, Verleger and colleagues have proposed that P3 reflects (re)activation of well-established S-R links^14,15^. In a typical laboratory task, usually a few fixed S-R links or S-R schemas are established by instruction and practice, e.g., “letter A → right hand” or “letter U → left hand”. Such a link binds a stimulus-code with its corresponding response-code, forming a ready-to-use schema – a sensorimotor event representation or an “event file”^16,17^ (cf. the related concept of affordance^18^, or the ideomotor principle^19^). Consequently, perceiving the matching stimulus automatically activates the corresponding, already well-established, motor program, and this process is assumed to be reflected by P3.

In the present study, we used a simple experimental task adapted from Verleger, Siller, Ouyang, and Śmigasiewicz^20^. Each trial of the task consisted of two successive visual stimuli, S1 and S2. S1 was a white letter A or U, and S2 was a color change of the white letter for blue or yellow. The task was divided into two experimental conditions (block-wise). In the first condition, participants had to indicate whether S2 was blue or yellow. We termed this condition “S1-irrelevant” or S1-I, because with S2 signaling both *how* and *when* to respond, S1 provided no response-relevant information. In the second condition, participants had to indicate whether S1 was the letter A or U, but only after S2 onset. Here, S1 provided all information on *how* to respond, while S2 signaled only *when* to respond, and the condition was termed S1-relevant or S1-R. The crucial experimental difference was that in the S1-irrelevant condition, the correct response program could be activated and executed only after S2 onset, whereas in the S1-relevant condition, the correct response could be selected and activated as soon as S1 was detected, but executed only after the “go” signal, so that the response (pre)activation and response execution were split apart. Importantly, if participants indeed extracted information already from the relevant S1 and activated the proper response program before S2 onset, their responses should be faster in the S1-R than in the S1-I condition.

If P3 is related to stimulus evaluation only, and not to response selection or activation (the stimulus evaluation hypothesis), then P3 will be evoked only by the target stimuli, i.e., S1 in the S1-R condition and S2 in the S1-I condition, and not by the go S2 (cf.^14^). Moreover, the amplitude of the target-evoked P3 will be similar in the both conditions. Alternatively, if P3 reflects activation of S-R links or event files (the S-R link hypothesis), the largest P3 will be evoked by S2 in the S1-irrelevant condition, i.e., when S2 works as both the target and go signal, because then the required S-R link is needed to be immediately fully activated and executed. Whereas in the S1-R condition, the target stimuli (the relevant S1) and the go signal (S2) are separated in time, so that when S1 is presented, participants have no time pressure to activate the S-R link, and have to even wait with its execution. Thus, based on previous results and revisions of the S-R link hypothesis^14,15^, we expected that in the S1-R condition, S1 will only initially pre-activate the corresponding S-R link, evoking some intermediate P3. Then, the go signal will reactivate the link again, evoking an “echo” of the first activation, i.e., another intermediate P3, enabling the actual execution of the prepared response program (cf ^14^). To control for the possibility that the S1 relevance effects on P3 might be confounded with other electrophysiological activity evoked before or simultaneously with P3, we also measured early visual evoked potentials (VEPs), a visual P2 component, and the Contingent Negative Variation (CNV).

### EEG methods

#### Participants

Twenty-seven students (20 females, mean age 21.7 years, SD 3.6) participated in the EEG experiment. Participants were paid 30 PLN (equivalent to €7). All participants were right-handed, had normal or corrected-to-normal vision, and had no history of neurological disorders. The study was approved by the local ethics committee at the Institute of Psychology of the Jagiellonian University, all experimental methods were carried out in accordance with guidelines and regulations, and a written informed consent was obtained before the experiment from each participant, in accordance with the Declaration of Helsinki.

#### Experimental task and procedure

**Figure 1:**
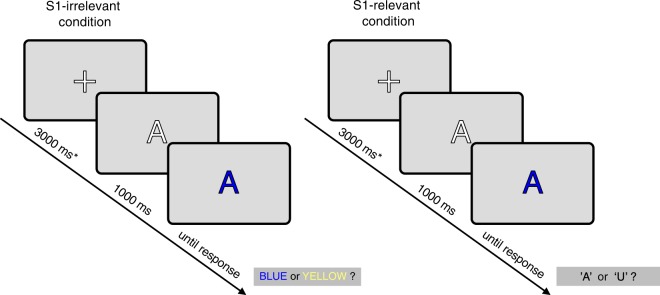
Illustration of the tasks and examples of the stimuli used in the EEG and fMRI experiments. The experimental procedure consisted of two experimental conditions: ‘S1-irrelevant’, and ‘S1-relevant’. In the S1-irrelevant condition, participants had to indicate whether S2 was blue or yellow. In this condition, S2 signaled both *how* and *when* to respond, while S1 provided no response-relevant information. In the S1-relevant condition, participants had to indicate whether S1 was the letter ‘A’ or ‘U’, but only after S2 onset (i.e. letter color change). Here, S1 provided all information on *how* to respond and S2 was only a go signal. See Methods for details. *In the fMRI experiment, the fixation interval was 6000 ms.

The task is illustrated in Fig. 1. The procedure and stimuli have been adapted from Verleger *et al*.^20^. Each experimental trial consisted of a fixation period followed by two successive stimuli, S1 and S2. Fixation point was a 1.5° high white ‘+’ (RGB: 255,255,255), and it was displayed for 3000 ms, so that the fixation period served also as an inter-trial interval and a baseline condition. S1 was a 1.5° high white capital letter ‘A’ or ‘U’ (RGB: 255,255,255), and it was presented for 1000 ms. After this interval, the displayed letter changed its color to blue or yellow (RGB: 0, 0, 255, and 255, 255, 0, respectively) - this color change constituted S2. Thus, S2 was the same letter as S1, but colored: it was a blue or yellow ‘A’, or a blue or yellow ‘U’. S2 also served as the “go” signal for response and was presented until participant pressed one of the response buttons. The next trial started automatically after the response. All stimuli were presented at the center of the computer screen (24’) on grey background (RGB: 220, 220, 220). The letters and colors were chosen randomly with equal probability for each alternative.

The task consisted of two experimental conditions: S1-irrelevant condition and S1-relevant condition. In the S1-irrelevant condition, participants had to indicate whether S2 was blue or yellow, by pressing the left button (using left thumb) for ‘blue’ or the right button (using the right thumb) for ‘yellow’ (counterbalanced between participants). In the S1-relevant condition, participants had to indicate whether S1 was the letter ‘A’ or ‘U’, but only after S2 onset, by pressing one button using the left thumb for ‘A’ or the other button using the left index finger for ‘U’ (response hand was changed block-wise, the whole response pattern was counterbalanced between participants). Therefore, in the S1-irrelevant condition, S2 signaled both *how* and *when* to respond, whereas in the S1-relevant condition, S1 signaled *how* to respond and S2 *when* to respond. Stimulus-wise, the two conditions were identical. Participants responded using a Cedrus RB-830 response box (Cedrus Corporation, San Pedro, CA). Presentation® software (Neurobehavioral Systems Inc., Albany, CA) was used for experimental control.

At the beginning of the session, participants were given written and then verbal instructions, and were also instructed to keep central fixation throughout the whole trial and to respond right after S2 onset. The task began with a practice session consisting of 20 trials, followed by 300 experimental trials divided into 4 blocks; Two blocks of the S1-irrelevant condition were followed by two blocks of the S1-relevant condition. The task was performed in this order to ensure that any learning effects from the S1-relevant condition had no impact on processing S1 in the S1-irrelevant condition. Additionally, in 20% of trials of each block, only S1 was displayed and did not require any reaction from participants (they had been informed about these no-S2 trials in the instruction). These control trials were included to discourage automatic, premature responses, and to allow accurate estimation of the haemodynamic response function (HRF) for S1 stimuli in the fMRI experiment. Each block consisted of 75 trials (60 trials with both stimuli, 15 trials with S1 only), which resulted in 150 trials per experimental condition. The order of trials within blocks was randomized for each participant. The task lasted up to about 40 minutes.

At the end of the session, participants were asked to rate their subjective experience of stimulus visibility, separately for S1 and S2 for both conditions, using a four-point Perceptual Awareness Scale or PAS developed by Ramsøy and Overgaard^21^, with the points labeled as follows: 1. ‘*No experience*’, 2. ‘*Brief glimpse*’, 3. ‘*Almost clear image*’, and 4. ‘*Absolutely clear image*’. The PAS was used here to identify any differences in subjective visibility of S1 and S2 stimuli in the two task conditions. Subjects read the instruction about the rating rules of PAS scale before the experiment.

#### Behavioral data analysis

Accuracy was analyzed using a non-parametric Wilcoxon signed rank test. To handle a substantial number of zero differences between paired conditions, scores in one condition were jittered randomly by a very small value and then a Wilcoxon test was applied. To acquire a reliable estimation of *p*-value, this procedure was repeated one million times, and then the mean *p*-value was calculated. We do not report false alarms because there was not a no-go stimulus in no-S2 trials – a fixation was displayed instead, indicating the beginning of a new trial – thus participants could not produce false alarms. Response times were analyzed using a repeated measure ANOVA with S1 Relevance (S1-irrelevant, S1-relevant) as within-subject factor.

#### EEG recording and preprocessing

BioSemi ActiveTwo system (Biosemi, Amsterdam, NL) equipped with Ag/AgCL active electrodes was used to acquire EEG data, stored with a 1024 Hz sampling rate. We used 64 scalp electrodes mounted on an Electro-Cap in accordance with the extended 10–20 system, and two additional electrodes, the common mode sense (CMS) active electrode and the driven right leg (DRL) passive electrode, as reference and ground electrodes, respectively (www.biosemi.com/faq/cms&drl.htm). The vertical electro-oculogram (EOG) was recorded from bipolar electrodes attached above and below the left eye and the horizontal EOG was recorded from the bipolar electrodes located at external canthi of both eyes.

BrainVision Analyzer software (version 2, Gilching, DE) was used for offline EEG data processing. Data were filtered with a 0.016–30 Hz band-pass filter (Butterworth zero phase filters, attenuation of 24 dB/octave) and 50 Hz notch filter. In the next step, data were re-referenced to linked mastoids, and split into non-overlapping segments for analysis of event-related potentials. Only epochs from trials with correct responses were included. All segments were 2000 ms long and included 200 ms before S1 onset, 1000 ms of S1-S2 onset interval, and lasted until 800 ms after S2 onset. A period of the first 200 ms of each segment was used for baseline correction. To ensure accurate ocular correction, trials with prominent artifacts present at the cephalic electrodes were removed first by rejecting epochs with overall minimum-maximum voltage differences exceeding 280 µV or with voltage steps between adjacent data points exceeding 60 µV. In the next step, blinks and eye movement artifacts were corrected using the Gratton–Coles method^22^ as implemented in the Brain-Vision Analyzer. Artifact-corrected data were baseline-corrected and edited for other artifacts by rejecting trials with overall minimum-maximum voltage differences exceeding 150 µV, or with absolute amplitudes exceeding 200 μV within segment. Artifact-free segments were averaged over each relevant condition separately for each participant. The average number of accepted segments was 101 (*SD* 16) for the S1-irrelevant condition, and 99 (*SD* 17) for the S1-relevant condition.

#### EEG measurement and analysis

**Figure 2:**
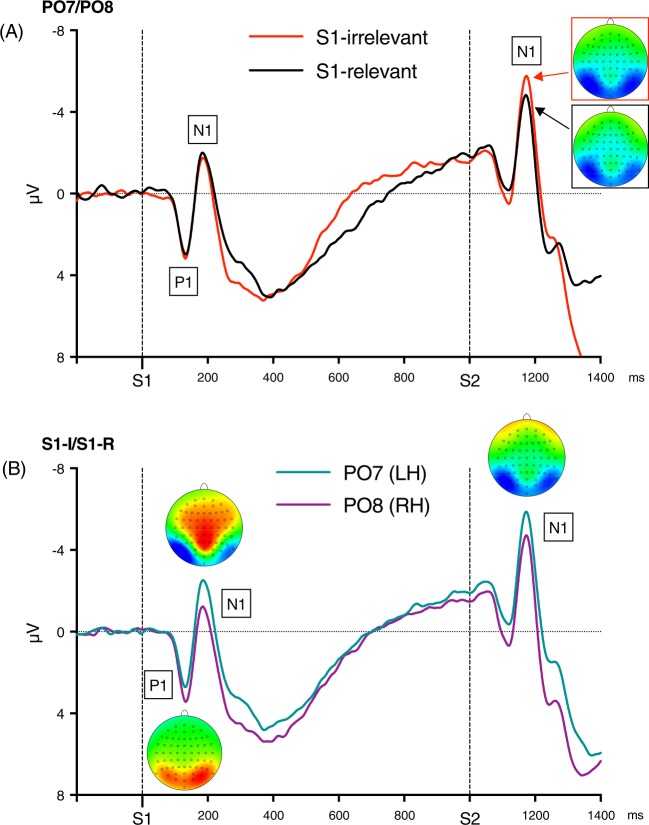
(**A**) Grand means of visual evoked potentials (VEPs) evoked by S1 and S2 in both experimental conditions (red line: S1-irrelevant condition, black line: S1-relevant condition) pooled across PO7 and PO8 sites. The head maps depict topographies of the S2-evoked N1 maxima. Colors of arrows and frames denote the experimental conditions. (**B**) Grand means of VEPs evoked by S1 and S2, presented separately for the left and right hemisphere, but pooled across the two task conditions. The head maps depict peak topographies of the S1-evoked P1 and N1, and the S2 evoked N1. Negative voltage points upwards. Time-point zero is S1 onset. The maps are min-max scaled, with positive polarity in red, negative polarity in blue. The head view is from above.

*Visual evoked potentials (VEPs)*. The VEPs formed regular waveforms with well-defined P1 and N1 components, which were measured in averages from PO7 and PO8 sites, where the peaks were largest (see Fig. 2). The S1-evoked VEPs were measured relative to pre-S1 baseline, as mean amplitude 120–140 ms for S1-evoked P1, and 180–220 ms for S1-evoked N1. The S2-evoked VEPs were measured peak-to-peak, N1 relative to the preceding P1, to bypass the issue of the baseline affected by the preceding S1-evoked activity (cf.^23^): S2-evoked P1 was measured as mean amplitude 120–140 ms after S2 onset, and N1 180–220 ms after S2 onset. The VEP amplitudes were submitted to repeated measures 2 × 2 ANOVAs with S1 Relevance (S1-irrelevant, S1-relevant) and Hemisphere (PO7, PO8) as within-subject factors.

**Figure 3:**
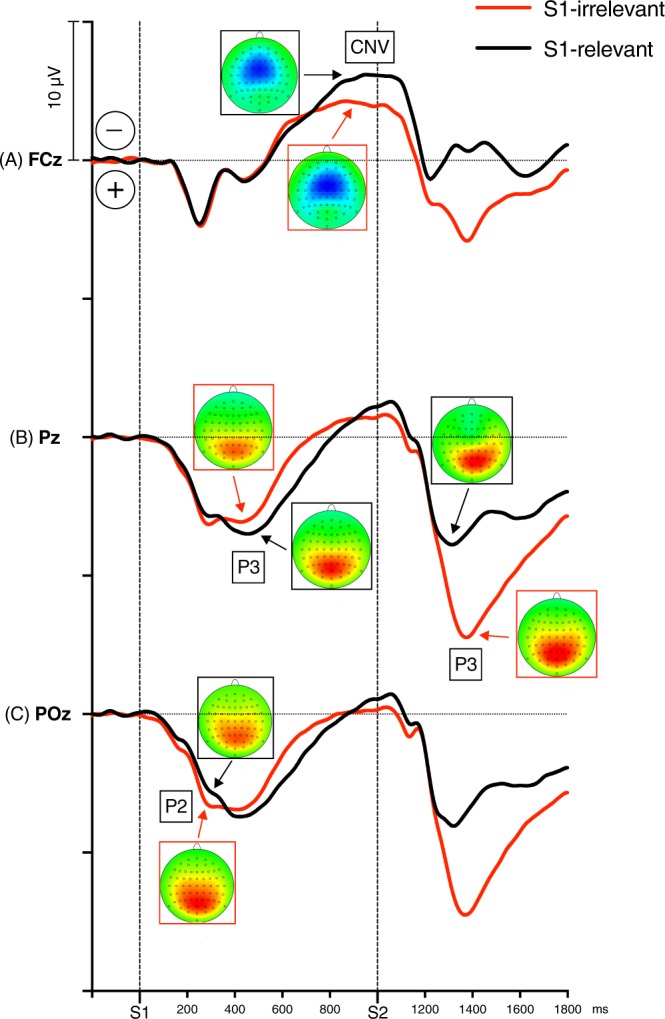
Grand means of ERPs evoked by S1 and S2 in both experimental conditions (red lines: S1-irrelevant condition, black lines: S1-relevant condition), recorded at FCz - to illustrate the CNV component (panel A), at Pz - to illustrate the S1- and S2-evoked P3 components (panel B), and at POz - to illustrate the S1-evoked P2 component (panel C). Negative voltage points upwards. Time-point zero is S1 onset. The head maps depict peak topographies of the ERP components indicated by the arrows. Colors of arrows and frames denote the experimental conditions. The maps are min-max scaled, with positive polarity in red, negative polarity in blue. The head view is from above.

*P*2 *component*. The S1-evoked P3 was preceded by a small positive component, which we classified as P2 and measured as mean amplitude 260–310 ms at Pz and POz sites, where it was largest (see Fig. 3B,C). P2 was not present in S2-evoked ERPs. The P2 amplitudes were tested by a repeated measures 2 × 2 ANOVA with the factors S1 Relevance (S1-irrelevant, S1-relevant) and Recording Site (Pz, POz).

*P3 component*. P3 was measured at Pz, where the relevant peaks were largest. S1-evoked P3 was measured as mean amplitude 360–560 ms after S1 onset, relative to pre-S1 baseline. S2-evoked P3 was measured as mean amplitude 260–560 ms after S2 onset, relative to mean amplitude 0–100 ms after S2 onset, to bypass the issue of different P3 starting points in the S1-irrelevant and S1-relevant conditions (shifted by S1-evoked activity; see Fig. 3B). The P3 amplitudes were submitted to repeated measures ANOVAs with the factor S1 Relevance (S1-irrelevant, S1-relevant). P3 latencies were not measured due to the entanglement of the P2 and P3 components in the S1-evoked ERPs.

*Contingent Negative Variation (CNV)*. A CNV component emerged between S1 and S2. It was measured at Cz, where it was largest, as mean amplitude 900–1100 ms after S1 onset (epoch determined based on the grand averages, see Fig. 3A), and was submitted to a repeated measure ANOVA with S1 Relevance (S1-irrelevant, S1-relevant).

### Results of the EEG experiment

#### Behavioral results

The average response accuracy was very high and similar in both experimental conditions: It was 99.0% (*SD* 1.3%) in the S1-irrelevant trials and 98.6% (*SD* 2.0%) in the S1-relevant trials, *p* = 0.15. The average response time was significantly longer in the S1-irrelevant trials (506 ms) than in the S1-relevant trials (451 ms), *F*_1,26_ = 20.17, *p* < 0.001, *η*_*p*_^2^ = 0.44. The ratings of perceptual awareness showed that all the stimuli were clearly visible, ranging from the third to fourth point of the PAS scale, and were perceived equally well in both experimental conditions. The PAS averages and ranges for each condition are shown in Table 1.

**Table 1:** Mean PAS ratings of S1 and S2 in the S1-irrelevant and S1-relevant conditions of the EEG and fMRI experiments.

		Mean rate and range	
Experiment	Stimulus	S1 Irrelevant	S1 Relevant
EEG	S1	3.85 (3–4)	3.85 (3–4)
	S2	3.88 (3–4)	3.77 (2–4)
fMRI	S1	3.52 (3–4)	3.59 (3–4)
	S2	3.63 (3–4)	3.56 (3–4)

#### EEG results

*Visual Evoked Potentials (VEPs)*. Grand-average of the VEPs averaged across the PO7 and PO8 sites are depicted in Fig. 2A, separately for the two relevance conditions. Figure 2B shows the grand-averages separately for the PO7 and PO8 sites, to illustrate differences between the hemispheres.

The S1-evoked VEPs were not affected by S1 Relevance, *F*s < 1.0. The N1 amplitude seems slightly larger in the S1-relevant condition (see Fig. 2A), but this trend was caused by the already emerging P2 component, which was larger in the S1-irrelevant condition (see the N1 head map in Fig. 2B and the P2 section below). The S1-evoked VEPs were, however, modulated by Hemisphere: The P1 amplitude tended to be larger at the right hemisphere, *F*_1,26_ = 3.6, *p* = 0.069, *η*_*p*_^2^ = 0.12, and the N1 amplitude was larger at the left hemisphere, *F*_1,26_ = 6.56, *p* = 0.017, *η*_*p*_^2^ = 0.20 (see Fig. 2B). The Hemisphere effects did not interact with Relevance (Hemisphere × Relevance for P1, *F* < 1.0, n.s., and for N1: *F*_1,26_ = 2.52, *p* = 0.12, *η*_*p*_^2^ = 0.08).

Unlike the S1-evoked VEPs, the S2-evoked VEPs were affected by S1 Relevance: The N1-P1 amplitudes were on average 1.5 *µV* larger in the S1-irrelevant than in the S1-relevant condition, *F*_1,26_ = 34.09, *p* < 0.001, *η*_*p*_^2^ = 0.57. Other effects were not significant, *F*s < 1.0.

*P*2 *component*. In the S1-evoked ERPs, P3 was preceded by a noticeable component peaking about 280 ms after S1 onset at parieto-occipital midline (Pz, POz; see Fig. 3B,C). We classified this component as P2. This P2 was noticeably better pronounced in the S1-irrelevant than in the S1-relevant trials. The difference between the two conditions was significant at POz, but not at Pz (main effect of Relevance: *F*_1,26_ = 4.87, *p* = 0.036, *η*_*p*_^2^ = 0.16; Relevance × Site interaction: *F*_1,26_ = 10.66, *p* = 0.003, *η*_*p*_^2^ = 0.29; Relevance at POz: *F*_1,26_ = 8.14, *p* = 0.008, *η*_*p*_^2^ = 0.24; Relevance at Pz: *F*_1,26_ = 2.20, *p* = 0.15, *η*_*p*_^2^ = 0.08). The P2 component was absent in the S2-evoked ERPs.

**Figure 4:**
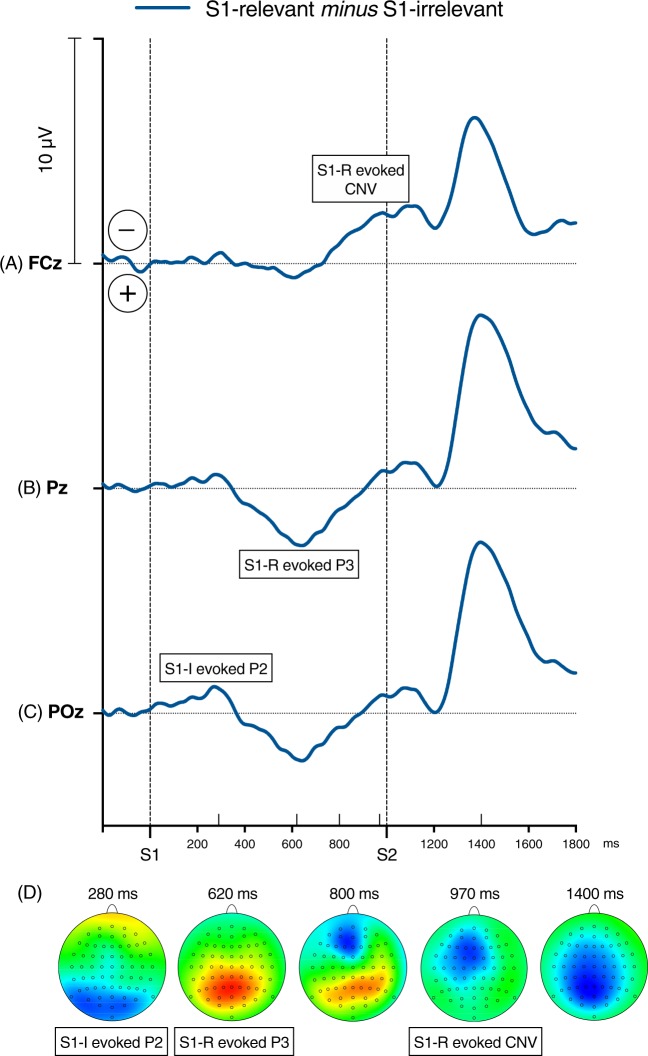
S1-relevant *minus* S1-irrelevant ERP difference waves recorded at (**A**) FCz, which shows the increase of CNV in the S1-R condition; (**B**) Pz – the positive peak between 600–700 ms after S1 onset indicates P3 evoked specifically by the relevant S1, and the negative peak between 300–600 ms after S2 onset reflects smaller S2-evoked P3 in the S1-R condition, i.e., when S2 was only the “go” signal; and (**C**) POz – the negative peak about 240–320 ms after S1 onset shows P2 evoked by the irrelevant S1. Negative voltage points upwards. Time-point zero is S1 onset. Panel D depicts topographies of the S1-relevant *minus* S1-irrelevant difference at the following time-points after S1 onset: 280 ms (S1-I-evoked P2), 620 ms (S1-R-evoked P3), 800 ms (P3-CNV overlap), 970 ms (S1-R-evoked CNV), and 1400 ms (S2-evoked P3). These time-points are denoted on the X-axis by the upward pointing ticks. The maps are min-max scaled, with positive polarity in red, negative polarity in blue. The head view is from above.

*P3 component*. Figure 3B depicts the S1- and S2-evoked grand average waveforms recorded at Pz, time-locked to S1, separately for the S1-irrelevant and S1-relevant conditions, along with P3 topographies. In the S1-evoked part of the ERPs, P3 is unequivocally larger in the S1-relevant condition than in the S1-irrelevant condition. In the S2-evoked ERPs, on the other hand, P3 is about twice as large in the S1-irrelevant trials than in the S1-relevant trials. Figure 4B shows the S1-R − S1-I difference wave. The difference between the conditions reached its maxima at about 620 ms after S1 onset, reflecting a larger and more prolonged P3 in the S1-R condition, and 400 ms after S2 onset, reflecting a larger and more prolonged P3 in the S1-I condition. In both cases, the difference peak was centered at Pz.

The ANOVA confirmed that the S1-evoked P3 was larger in the S1-relevant condition than in the S1-irrelevant condition, *F*_1,26_ = 5.79, *p* = 0.024, *η*_*p*_^2^ = 0.18. The S2-evoked P3 displayed the opposite pattern, showing significantly larger P3 amplitudes in the S1-irrelevant trials than in the S1-relevant trials, *F*_1,26_ = 73.91, *p* < 0.001, *η*_*p*_^2^ = 0.74.

*CNV component*. The CNV component had typical midfrontal distribution with the negative maximum at FCz about 900–1100 ms after S1 onset (depicted in Fig. 3A). The CNV amplitude was significantly larger in the S1-relevant condition than in the S1-irrelevant condition, *F*_1,26_ = 20.96, *p* < 0.001, *η*_*p*_^*2*^ = 0.45.

### Discussion of the EEG experiment

#### Behavioral results

Participants had the same near-ceiling accuracy rate in both experimental conditions, while the responses were markedly faster in the S1-relevant condition than in the S1-irrelevant condition. This appears to confirm that in the S1-R condition participants indeed extracted information already from S1 and activated the correct S-R link before onset of the go signal. Moreover, participants reported equal subjective visibility of S1 and S2 regardless of S1 relevance, indicating that the relevant and irrelevant S1s were perceived with the same subjective stimulus awareness.

#### ERP results

The obtained ERP results displayed a typical pattern of consecutive VEP, P3, and CNV components that closely resembles findings from previous studies^3,24,25^, thereby providing a solid ground for interpretation of the observed experimental effects.

*Visual evoked potentials (VEPs)*. The relevance manipulation had no effect on the S1-evoked VEPs, which indicates that at the perceptual level both the relevant and irrelevant S1s were processed alike, and conforms to the S1 accuracy and subjective visibility measures. It is well known that visual attention, often guided by stimulus relevance, modulates the VEP amplitudes^26^. Accordingly, the obtained results suggest that the participants paid attention to the first stimulus in a trial regardless its relevance (possibly, their attention was captured by a relatively salient S1), thus it seems that the following relevance effects on P3 are not attention-related artifacts. Unlike the S1-evoked VEPs, the S2-evoked VEPs were slightly larger in the S1-irrelevant condition (i.e., when evoked by the target S2) than in the S1-relevant condition (i.e., when evoked by the go S2), indicating stronger responses of visual system when the stimuli were targets and go signals at once than go signals alone. The latter result may be explained as a top-down attentional modulation^27^.

Furthermore, the S1-evoked VEPs displayed a typical pattern of hemispheric asymmetry. The P1 component tended to be larger at the right hemisphere (RH), in line with evidence for a RH advantage at early cortical stages of perceptual processing^28,29^. The N1 component was in turn larger at the left hemisphere (LH), in line with evidence for localization of the visual word form area in the left lateral occipito-temporal sulcus^30^ and recent EEG evidence showing that single letters, as whole words, evoke larger N1 component over the left occipital cortex^31^. No hemispheric asymmetry was found in the S2-evoked VEPs, suggesting no lateralization of color processing.

*P2 component*. In the S1 evoked ERPs, the VEPs were followed by a small component peaking about 280 ms after S1 onset over parieto-occipital areas, which we classified as P2. Interestingly, this S1-evoked P2 was larger in the S1-irrelevant condition; in fact, P2 was hardly present in the S1-relevant condition. The functional meaning of this component is poorly understood^32^. However, there is evidence (from studies using auditory stimuli) that non-target stimuli evoke larger P2 than target stimuli^33,34^, and that an increased attention to stimuli may decrease the P2^35^. Thus, it has been suggested that P2 may reflect a process of identification of a stimulus as a target, and that P3 emerges if the target had been detected^33,35^. This interpretation apparently fits well with the present results (see below). More recently, a visual-evoked parieto-occipital P2, much alike the current one, has been found in a priming task and linked to a suppression mechanism^36^, which also may account for the present results. In agreement with this interpretation, no P2 component was found in the S2-evoked ERPs, because S2 was always relevant.

**Figure 5:**
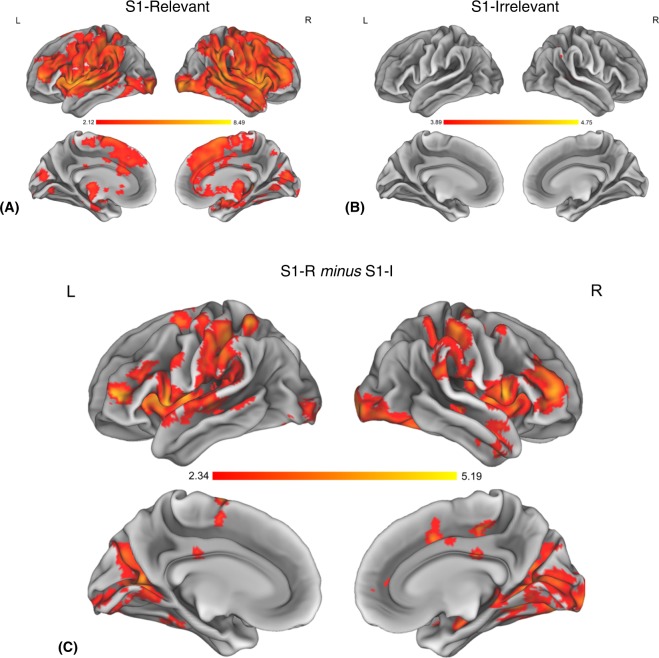
Panels (A,B) show activations for the main effects in the S1-I and S1-R conditions, respectively. Visible clusters of activity are associated with processing of the S1 stimulus. Panel (C) shows results of the Whole-Brain Mass-Univariate analysis for the S1-R *minus* S1-R contrast. Suprathreshold clusters represent brain regions associated with increased processing of the S1 stimulus in the S1-R conditions. The shades of red represent values of *t*-statistic. L = left hemisphere, R = right hemisphere.

*P3 component*. In the S1-irrelevant condition, a relatively small S1-evoked P3 was followed by a large and unambiguous S2-evoked P3. Whereas in the S1-relevant condition, both the target S1 and the following go S2 evoked moderate P3s. Moreover, the S1-evoked P3 was smaller in the S1-irrelevant condition than in the S1-relevant condition. This result pattern resembles the results observed in studies using the oddball task (see the P3s evoked by rare stimuli in: Verleger *et al*.^14^, Fig. 2, SOA 400, p.275; Verleger *et al*.^15^, Fig. 5, p.32; Verleger *et al*.^20^, Fig. 3, SOA 300, 400, 500, p.6).

These results may therefore be interpreted in terms of the S-R link activation hypothesis of P3. The target S2 (S1-I condition) evoked largest P3, because then the S-R link (corresponding with S2) had to be immediately fully activated and executed. The target S1 and the go S2 stimuli (S1-R condition) evoked in turn about half as large P3s. In this case, although S1 provided all information about the response, participants had to postpone the response until the go signal appeared. Thus, the corresponding S-R link was initially pre-activated with S1 onset, allowing to achieve an optimal response readiness, and then re-established after the go signal, thereby putting the S-R codes through to the actual response execution (cf.^14,15^). However, the S-R link hypothesis also predicts that the irrelevant S1 would evoke no P3, because this stimulus was not associated with any task-related S-R link. Yet, although smallest, P3 was still present in this condition. One explanation is that the S-R hypothesis is simply incorrect. Still, considering that S1 was a letter, one may argue that a small P3 should be in fact evoked by the irrelevant S1. It is well known that letters are read automatically, even when reading is task-irrelevant or counter-productive (cf. the Stroop effect^37^), and letter processing recruits automatically the whole sensorimotor reading network^38^. It is therefore arguable that perceiving a letter automatically pre-activates the corresponding S-R link, i.e., an established (through a lifelong practice) binding of perceptual and reading (motor) codes. Future studies might examine this issue by comparing letters to other stimuli, which should be ﻿unfamiliar to participants and difficult to verbalize, e.g., Tibetan letters^28^ or irregular geometric shapes^39^.

It would be more difficult to explain the obtained P3 results in terms of the stimulus evaluation account. The large difference between the target-evoked P3s in the S1-I and S1-R conditions, and similar magnitude of the S1- and S2-evoked P3s in the S1-relevant condition argue against this hypothesis, which predicts that similar P3 would be evoked by both targets and that P3 would be evoked only by the target stimuli and not by the go signal (cf.^14^). The development of the idea that P3 is related to stimulus-related processes and is independent of response selection began with the seminal study by Kutas and colleagues^9^ demonstrating a positive trial-by-trial correlation between RT and P3 latency under condition in which response generation was tightly coupled with stimulus evaluation. This finding has been followed by a body of evidence from various experimental paradigms^7,40,41^. For example, McCarthy and Donchin^6^ have demonstrated that while RT was affected by manipulations of stimulus discrimination and S-R compatibility, P3 latency was affected only by the former, indicating that P3 is not sensitive to S-R coupling. In their Stroop study, Duncan-Johnson and Kopell^42^ have found that while RT was affected by the Stroop response interference, P3 latency remained constant across the congruent and incongruent condition, again in line with the assumption that P3 indexes only stimulus processing, and not response processing. More recently, Twomey *et al*.^43^ analyzed data from three experiments using auditory oddball tasks and visual target detection task, and found that P3 latency varied systematically with onsets of a build-to-threshold perceptual decision, also suggesting that that “*the peak latency of the P300 indexes the duration of stimulus evaluation processes*” (p.1641). The stimulus evaluation hypothesis has been however increasingly often contradicted by findings that P3 latencies are also affected by factors related to response processing. A review by Verleger^41^, including 143 data sets from fifteen experimental paradigms, showed that the postulated relationship between RT and P3 latency highly varied between the experimental paradigms. While in some tasks the RT-P3 relationship was linked to stimulus-related processing, in others, like the Simon task and the flanker task, this relationship reflected predominantly response selection processes. Moreover, when experimental procedures are specifically design to examine the relationship between P3 and S-R coupling, the results rather systematically contradict the assumption of the stimulus evaluation account that P3 is not related to response processing^14,15,20,44^. Thus, the assumption seems unsustainable (cf.^45^). Still, more studies are needed to determine what exactly is reflected by P3, since it is also possible that it may represent a bundle of parallel yet functionally distinct processes taking place in the same time window.

*CNV component*. The contingent negative variation (CNV) is a large centro-parietal ERP component reflecting processes of anticipatory attention and motor preparation following e.g., an accessory stimulus announcing the arrival of an imperative stimulus^24^. We found this component to be significantly larger in the S1-relevant condition than in the S1-irrelevant condition. Above, we have suggested that the relevant S1 elicits pre-activation of S-R link, allowing to achieve an optimal response readiness while waiting for the go signal. This interpretation appears to be supported by this larger CNV in the S1-relevant condition: The CNV is large here because the required response is already fully prepared, whereas in the S1-irrelevant condition only temporal preparation is possible.

Notwithstanding, the presence of CNV is an issue for our interpretation of the effect of S1 relevance on P3. As seen in Fig. 3A, the CNV component emerges already at about 550 ms after S1 onset, partially overlapping with P3. Therefore, the larger positivity in the S1-relevant condition might actually be a larger negativity in the S1-irrelevant condition, assuming that CNV had earlier onset in this condition. Inspection of the P3 and CNV time-course in the two conditions and the topography of the S1-R – S1-I difference (see Fig. 4) suggest that this does not seem to be the case here. Moreover, we found that the S1-R-evoked P3 was neither correlated with the S1-I-evoked CNV (*r* = 0.07, *p* = 0.72) nor the S1-R-evoked CNV (*r* = 0.18, *p* = 0.36), which also suggest that the S1-R-evoked positivity was not a confound of the S1-evoked negativity. This conclusion is in line with Verleger *et al*.’s^20^ evidence against a major confound of P3 by CNV overlap. Although there is an overlapping time-window, it seems that the 1 sec long target – go signal interval was long enough to mostly separate the peak activities of the two components. Still, to prove this we would have to dissociate them experimentally (e.g., by the oddball manipulation; cf.^20^).

#### Summary of the EEG experiment

The aim of the EEG experiment was to investigate whether P3 is related to stimulus evaluation only, or also to response processing. The obtained results suggest that P3 may be related to processes of coupling stimulus and response codes, and might be interpreted in terms of the hypothesis of S-R link activation. Still, further studies are needed to disentangle P3 from CNV, and to examine whether the irrelevant S1 evoked P3 indeed because S1 was a letter.

## fMRI EXPERIMENT

### Introduction

Our tentative conclusion from the EEG experiment is that P3 may reflect activation of established S-R links or event files. Importantly, after subtracting the S1-irrelevant from the S1-relevant condition, all of the remaining S1-evoked activity may be interpreted as neural underpinnings of the activation of S-R links or sensorimotor event files, and all of the remaining S1-evoked positive activity may be classified as the P3 component related to this S-R link activation. As EEG and fMRI signals have a common neuronal source (i.e., local field potentials^46^), in the fMRI experiment, we focused on the S1-R *minus* S1-I contrast related to the S1-evoked activity, aiming at identifying the brain areas and connectivity underlying these sensorimotor links. We used the same task as in the EEG experiment. Differences between the S1-relevant and S1-irrelevant conditions in the S1-evoked fMRI activations were tested by means of standard whole-brain mass-univariate analysis. To test the differences between the two conditions in brain functional connectivity, the fMRI data were analyzed using the network-based statistics approach^47^, which allows to identify clusters of connections that differ between task conditions and thus to delineate condition-related changes of the strength of connections between relevant cortical regions.

Neuroimaging studies of event files have been sparse. The available evidence demonstrates that perceiving of only one feature or component of a well-established sensorimotor event file automatically activates neural representation of the whole unit^48–51^. For example, when a particular sensorimotor event file has been formed by bounding an image of a face with a right-hand response, then seeing this face again would activate both the corresponding neural representation of this sensory feature in the fusiform face area, and the corresponding neural representation of the response code bound to this sensory feature in the pre-motor and motor areas of the left hemisphere^50,51^. There is also evidence for involvement of the dorsolateral prefrontal cortex (DLPFC) in the managing the sensorimotor event files^52^. Based on these findings, we expected that, if the relevant S1 entails activation of established event files (or S-R links), the S1-R *minus* S1-I contrast will show the activation of the distributed sensorimotor network, including supplementary motor, premotor, and primary motor areas; parietal and prefrontal association areas, and plausibly also the dorsolateral prefrontal executive area, in addition to areas related to visual processing. Also, we expected that activation of this sensorimotor network will be reflected by an increased strength of large-scale connectivity within the whole network.

Having the results of the S1-R *minus* S1-I contrast, we will attempt to answer the question of whether the observed activity may be interpreted as the neuronal source of P3. Thus far, the neural network underlying P3 has not been well localized. The available lesion, intracranial, and imaging evidence suggest that visual-evoked P3 is generated by a network including higher visual areas in the inferior temporal cortex (IT), and supramodal association and attentional areas in the inferior parietal lobe (IPL, including the temporo-parietal junction, TPJ) and the posterior parietal cortex (PPC)^53–57^. These results appear to conform to the stimulus evaluation account, as reflecting the processes of allocation of perceptual and attentional resources to stimulus processing, and not related to any response processing. However, in most of these studies, experimental procedures (usually a variant of oddball task) were not well suited to answer the question whether P3 is related to processes linking stimulus and response. There appears to be no fMRI study investigating the neural generators of P3 interpreted as activation of S-R links or event files. The present study allows us to make a first step in this direction.

### fMRI methods

Only differences from the EEG experiment will be described.

#### Participants

Thirty-two volunteers (15 females, mean age 25.7, SD 3.1) participated in the fMRI experiment. None of them participated in the EEG experiment. One participant was excluded from the analysis due to extensive movement during two scanning sessions defined as having more than one time-point with framewise displacement larger than 3 mm^58^. The study was approved by the local ethics committee at the Institute of Psychology of the Jagiellonian University, all experimental methods were carried out in accordance with guidelines and regulations, and a written informed consent was obtained before the experiment from each participant, in accordance with the Declaration of Helsinki.

#### Experimental task and procedure

Minor changes were introduced to adjust the procedure for fMRI. The inter-trial time was increased from 3000 ms to 6000 ms. The stimuli were delivered through Visual System MRI compatible goggles (NordicNeuroLab AS, Bergen, NO) with a separate display for each eye, 800 ×600 pixels screen resolution, 32 bits color depth, and 85 Hz refresh rate. Participants responded using two ResponseGrip devices (NordicNeuroLab AS, Bergen, NO), one for each hand, containing two buttons per grip (for thumb and index finger). Participants were given written and then verbal instructions, and performed 20 practice trials before entering the scanner. Four fMRI scanning runs were performed, corresponding to the four blocks of the EEG experiment. The scanning session lasted up to about 50 minutes. The PAS was completed after the scanning session (PAS was assessed in 27 participants). All other task and procedure details were the same as in the EEG experiment.

#### fMRI data acquisition and preprocessing

fMRI data were acquired on a 3T GE Discovery MR750 scanner at the Centre for Modern Interdisciplinary Technologies, Nicolaus Copernicus University (Toruń, Poland). Each participant participated in two separate runs per condition during a single scanning session. Each run consisted of 340 volumes, except for three runs in three different participants that consisted of 335, 332, and 330 volumes, respectively. Functional data were acquired using a gradient-echo echo-planar imaging sequence (TR = 2 s, TE = 30 ms, FA = 90°, FOV = 192 cm, matrix size = 64 × 64, voxel size = 3 × 3 × 3 mm, no interslice skip, 42 slices). A high-resolution T1-weighted structural scan was also acquired from each participant (TR = 8,22 ms, TE = 3,19 ms, FA = 12°, FOV = 256 cm, matrix size = 256 × 256, voxel size = 1 × 1 × 1 mm, 256 slices).

Data preprocessing and further analysis was performed with FSL 5.0 software (http://fsl.fmrib.ox.ac.uk/fsl/fslwiki/FSL ^59–61^). Preprocessing steps involved automatic brain extraction using Brain Extraction Tool (BET, implemented in FEAT, FMRI Expert Analysis Tool), motion correction with MCFLIRT (implemented in FEAT), and spatial smoothing by Gaussian kernel with full-width at half-maximum of 6 mm. Motion artifact were filtered out using the ICA-AROMA toolkit (ICA-based Automatic Removal of Motion Artifacts^62^). ICA-AROMA uses independent component analysis to identify components showing motion related features, and it has been found to reduce motion artifacts and increase sensitivity to activations in task-based designs^62^. We used ICA-AROMA instead of other widely used approaches, e.g., 6 or 24 motion parameters regression, or motion-censoring, because it has been shown that ICA-AROMA performs better than motion regression and does not lead to decrease of temporal degrees of freedom^62,63^. Moreover, with our design, “motion censoring” could lead to different number of removed time-points in the two experimental conditions introducing bias to the analysis of differences between the two conditions. Lastly, the data were high-pass filtered with a cut-off period of 128 s and normalized to MNI standard space. First, the functional data were aligned to the high resolution T1 image using the Boundary-Based Registration method (implemented in FEAT), and subsequently registered to the MNI152 2 mm template using affine transformations with 12 degrees of freedom.

#### Whole-brain mass-univariate analysis

*Subject-level analysis*. A standard mass-univariate analysis was performed in the General Linear Model (GLM) framework (as implemented in FEAT). All trials with correct responses were divided into two sets, grouping S1 event and S2 event onset data respectively. Trials with incorrect responses and trials with no responses were grouped in a third set. These three sets of event onsets were used to create regressors of interest for GLM analysis. Even though it might be advantageous to model the exact duration of process of interest^64^, in our design matrix, we chose to represent all events as impulse functions of 1 second duration, since we did not know the temporal properties of the measured neural processes. As our procedure involved presenting two stimuli in close temporal proximity, we used partial trials and the basis function approach^65,66^ to correctly disentangle S1- and S2-related activity. Thus, using no-S2 trials in our design (20% of all trials) allowed to de-correlate the timing of S1 and S2 onsets, thereby reducing collinearity between regressors. Use of the basis functions instead of predefined hemodynamic response functions (HRF) allows for a more flexible modelling^67^, which is important for a reliable parameter estimation when there are multiple consecutive-events occurring in close temporal proximity in event-related designs^68^. For this purpose, we used the FLOBS method (FMRIB’s Linear Optimal Basis Sets, implemented in FEAT). Each event representation was convolved with three default FLOBS waveforms modelling possible temporal and dispersion shifts. As a result, three regressors were created for each event type (i.e., for S1, S2, and for trials with incorrect or missed responses). Therefore, three parameter estimates per each experimental event were calculated in the GLM estimation, decreasing error in the subject-level model fit. In order to provide an easily interpretable analysis at the group-level, only the first parameter estimate (which modelled the canonical HRF) was passed to the higher-level analysis. The benefit of this mixed approach is accounting for HRF variability, thus reducing error in the single-subject GLM fit, with multi-subject analysis remaining easily interpretable in terms of utilized statistics.

*Group-level analysis*. Before group-level analysis, parameter estimates from the two runs belonging to one condition were averaged. All group-level analyses were conducted with the RANDOMISE tool, which provides nonparametric permutation inference in FSL^69^. We used this method, because the recent study by Eklund, Nichols, and Knutsson^70^ demonstrated that only permutation-based inference ensures proper control of false positives in fMRI analysis. To test between-condition differences in S1-related activations, we used paired *t*-tests with 5000 random permutations. Calculated statistical maps were corrected for multiple comparisons (*p* < 0. 05, FWE) using threshold-free cluster enhancement (TFCE^71^). This method eliminates the need to arbitrarily specify cluster-defining threshold, and is sensitive to both spatial extent of the activation and strength of the signal. For illustration purposes, each statistical map was thresholded using a suitable arbitrary threshold in order to ‘divide’ the resulting statistical parametric images into separate clusters of activity. Note that TFCE enhances cluster-like structures, yet the statistical map remains voxel-wise, with every voxel having ascribed statistic value, i.e., the map is not divided into clusters.

#### Functional connectivity

*Beta-series correlation*. To examine differences between the two task conditions in functional connectivity, we tested whole-brain connectivity changes. We chose as seed regions-of-interests (ROIs) the set of 264 nodes provided by Power *et al*.^72^, which was based on a meta-analysis of activation studies. The ROIs were created as five-millimeter radius spheres centered around the coordinates listed in that publication. A beta-series correlation analysis^73^ was used to estimate effects of experimental manipulation, since it has been shown that beta-series have greater sensitivity in fast event-related designs than the psychophysiological interaction (PPI) approach^74^ – the second most popular method of estimating task-based connectivity. Usually, in beta-series analysis, one GLM model is constructed comprising as many regressors of interest as there are trials in the task, as a result yielding one parameter estimate for each trial. When the inter-stimulus interval (ISI) is short, as it was in the present study, this approach may result in collinear regressors inflating the variance of the estimated parameters^75,76^. Therefore, we utilized a modification of the ‘standard’ beta-series approach^75,77^, in which collinearity is reduced by using a separate GLM model for each trial, with the first regressor for the trials of interest, and the second regressor representing the remaining trials. Thus, in our analysis, each GLM model included three regressors: the first regressor for the S1 event of interest, the second regressor representing all remaining S1 events from the remainder of trials, and the third regressor for all S2 events. To reliably disentangle responses to adjacent events, we used the FLOBS basis sets method (similarly to mass-univariate analysis). Therefore, three final regressors were created for each original regressor. After estimation of all GLMs, parameter estimates for all events were sorted, and parameters for trials with incorrect and missed responses were excluded from further analysis. Parameter estimates for S1 events (including S1-only trials) were concatenated and formed the final beta-series. In each seed ROI, beta-series from all voxels were averaged to produce one beta-series per ROI. Beta-series from all ROIs were then correlated to produce a 264 × 264 correlation matrix. Finally, the correlation matrices from the two functional runs were averaged to create a single matrix per participant for each condition.

*Network-based statistics (NBS)*. The Network-Based Statistic Connectome toolbox (https://www.nitrc.org/projects/nbs/47) was used to identify clusters of S1-related functional connections that significantly differ between the two task conditions. Network-Based Statistics (NBS) were calculated on the weighted and unthresholded 264 × 264 functional connectivity matrices. To examine the S1-related between-condition differences, paired *t*-tests were calculated in a mass univariate manner, separately for each link connecting a pair of nodes. A threshold of *t* = 3.4 was used and only connections with *t* values exceeding that threshold were included in further analysis. Supra-threshold links were then organized in topological space into clusters of links for which a connecting path can be found between any two nodes. Finally, permutation testing with extent-based correction for multiple comparisons was used. 10,000 iterations were performed to generate a null distribution of the largest cluster size. The obtained subnetworks were considered as significant at family-wise error rate (FWER) corrected *p* < 0.05. All nodes were grouped into 7 anatomical divisions: frontal, insula, temporal, parietal, occipital, subcortical and cerebellum. Each ROI was assigned to one of the anatomical divisions based on the ROI coordinate in MNI space. For this purpose, an Automated Anatomical Labeling solution^78^ (as implemented in brainSpy tool) was used. In case of ambiguous locations corrections were made by hand based on the Harvard-Oxford Cortical and Subcortical Atlases and the Juelich Histological Atlas (as implemented in FSLeyes viewer). The list of anatomical designations of all nodes, MNI coordinates, and original Power *et al*.’s^72^ ROI numbers are presented in Table S1 in the Supplementary Information. The NBS results were visualized with the BrainNet Viewer (http://www.nitrc.org/projects/bnv/) and Circos^79,80^ (http://circos.ca/).

### Results of the fMRI experiment

#### Behavioral results

The average response accuracy was again very high and similar in both experimental conditions; the S1-irrelevant condition: 98.4% (*SD* 2.1%), the S1-relevant condition: 97.5% (*SD* 4.2%), *p* = 0.28. As in the EEG experiment, the average response time was longer in the S1-irrelevant condition (495 ms) than in the S1-relevant condition (403 ms), *F*_1,32_ = 17.49, *p* < 0.001, *η*_*p*_^*2*^ = 0.35. The perceptual awareness ratings showed that all the stimuli were very well visible, ranging from the third to fourth point of the PAS scale, and perceived equally well in both experimental conditions. The PAS averages and ranges for each condition are shown in Table 1. The results obtained in the fMRI experiment were therefore fully consistent with the EEG experiment.

#### fMRI results

*Whole-brain mass-univariate analysis.* The renderings representing the results of main effects for the S1-R and S1-I conditions separately are presented in Fig. 5 (panel A and B). In the S1-R condition, we have observed widespread activations encompassing prefrontal, premotor, and motor cortices, as well as strong activations in the peri-Rolandic areas in both hemispheres including the frontal pole, superior, middle and inferior frontal gyrus, precentral and postcentral gyrus. Extensive activations were present in the parietal lobe, including bilaterally areas in the angular gyrus, supramarginal gyrus, and superior parietal lobule. Significant activity was also found bilaterally in the insulo-opercular complex, the occipito-parietal, and occipito-temporal regions, including the lateral occipital cortex, occipital pole, cuneal cortex, intracalcarine cortex, lingual gyrus, and fusiform cortex. Further, activations were present bilaterally in the superior and middle temporal gyrus, and portions of inferior temporal gyrus, as well as in the medial areas including paracingulate and cingulate gyrus, and in the subcortical structures including hippocampus, pallidium, putamen and thalamus. The full list of clusters with coordinates is presented in Table 2. Activations present in the S1-I condition were constrained to two sites: angular gyrus, and nearby areas in the superior and middle temporal gyrus. The list of clusters with coordinates is presented in Table 3.

**Table 2:** Whole-Brain analysis results for the main effect of S1-Relevant condition, time-locked to the onset of S1 stimulus. Reported clusters are result of thresholding statistical map of significant t-values with an arbitrary threshold of t = 4.6. Only clusters larger than 10 voxels are reported. Reported results present areas with highest statistic values and coordinates of peak statistics. Reported clusters and associated voxel counts do not represent the full extent of significant results. Coordinates are reported in MNI space.

Hemisphere	Label	Voxels*	Max t (t > 4.6*)	X	Y	Z
Left	inferior frontal gyrus, superior temporal gyrus, central opercular cortex, frontal operculum, insula, putamen, pallidum	2279	7.22	−56	−2	2
Right	inferior frontal gyrus, superior temporal gyrus, central opercular cortex, frontal operculum, insula, putamen, pallidum	2162	8.49	48	4	−2
Right	supramarginal gyrus, angular gyrus, superior temporal gyrus, middle temporal gyrus	1048	7.49	50	−36	8
Right	supramarginal gyrus, angular gyrus, postcentral gyrus	320	5.81	54	−40	50
Right	middle frontal gyrus, frontal pole	260	5.61	36	42	20
Right	occipital pole, lateral occipital cortex	210	6.27	30	−96	−4
Right	precentral gyrus, middle frontal gyrus	92	6.47	58	8	40
Right	superior frontal gyrus	66	5.59	2	−2	68
Right	postcentral gyrus, precentral gyrus	54	5.14	60	−16	24
Right	middle temporal gyrus, temporal pole	51	5.86	44	10	−34
Left	occipital pole, lateral occipital cortex	50	6.09	−34	−94	−2
Left	frontal pole	46	5.68	−36	50	8
Left	cerebellum	39	5.57	−14	−58	−26
Right	postcentral gyrus, precentral gyrus	37	5.98	64	−2	20
Right	lateral occipital cortex	29	5.40	48	−78	−2
Left	hippocamus	19	5.34	−26	−14	−12
Left	supramarginal gyrus, parietal operculum	17	4.96	−56	−42	28
Right	precentral gyrus	15	5.47	34	−8	70
Left	planum temporale, superior temporal gyrus	14	5.48	−60	−40	18
Right	middle frontal gyrus, precentral gyrus	13	5.06	34	6	62
Left	superior parietal lobule, supramarginal gyrus, postecentral gyrus	13	4.99	−46	−40	56
Right	middle temporal gyrus, inferior temporal gyrus	12	5.16	66	−30	−18
Left	cerebellum	10	5.00	−10	−44	−24
Right	cerebellum	10	5.21	16	−40	−22
Left	angular gyrus, supramarginal gyrus	9	4.99	−52	−46	36
Right	cerebellum	7	5.14	2	−56	−14
Right	precentral gyrus, postcentral gyrus, middle frontal gyrus	6	5.13	40	−4	46
Right	cerebellum	6	5.16	12	−64	−20

**Table 3:** Whole-Brain analysis results for the main effect of S1-Relevant condition, time-locked to the onset of S1 stimulus. Reported clusters are result of thresholding statistical map of significant t-values with an arbitrary threshold of t = 3.9 Only clusters larger than 10 voxels are reported. Reported results present areas with highest statistic values and coordinates of peak statistics. Reported clusters and associated voxel counts do not represent the full extent of significant results. Coordinates are reported in MNI space.

Hemisphere	Label	Voxels*	Max t (t > 3.9*)	X	Y	Z
Right	angular gyrus, supramarginal gyrus, superior parietal lobule	118	4.43	44	−42	46
Right	superior temporal gyrus, middle temporal gyrus	116	4.75	56	−40	8

The full extent of activations for the S1-R *minus* S1-I contrast is presented in Fig. 5C. This contrast showed widespread activations related specifically to processing of the relevant S1. The most extensive activations in the frontal lobes were found in prefrontal and premotor cortices, as well as peri-Rolandic regions. In those regions bilateral suprathreshold activity was observed in the frontal pole, middle and inferior frontal gyrus, precentral and postcentral gyrus, as well as superior frontal gyrus on the medial surface (supplementary motor area). In the parietal lobe, activity was found in the supramarginal gyrus and the superior parietal lobule. Significant activations were also found bilaterally in the insulo-opercular complex, and in the occipito-parietal and occipito-temporal areas including the lateral occipital cortex, occipital pole, cuneal cortex, intracalcarine cortex, and lingual gyrus, further extending into fusiform cortex. Sites of significant activity were also observed bilaterally in the superior and middle temporal gyrus and posterior cingulate cortex and in the right hemisphere in the paracingulate gyrus. Smaller sites of activations were present in subcortical structures including the hippocampus in the right hemisphere, and bilaterally the pallidum. Significant activations were also present in both hemispheres of the cerebellum. The full list of clusters with coordinates is presented in Table 4.

**Table 4:** Results of the Whole-Brain analysis for the S1-R *minus* S1-I contrast, time-locked to the onset of S1. Reported clusters are results of thresholding the statistical map of significant t-values with an arbitrary threshold of t = 2.9. Only clusters larger than 20 voxels are reported. Therefore, reported clusters and associated values do not represent the full extent of significant results. Coordinates are reported in MNI space.

Hemisphere	Label	Voxels*	Max t (t > 2.9*)	X	Y	Z
Right	frontal pole, middle frontal gyrus, precentral gyrus, inferior frontal gyrus, frontal operculum, insular cortex	1954	5.07	24	50	6
Right	lingual gyrus, intracalcarine cortex, cuneal cortex, occipital pole, lateral occipital cortex, occipital fusiform cortex, temporal occipital fusiform cortex	1909	5.19	30	−48	−2
Left	middle frontal gyrus, precentral gyrus, inferior frontal gyrus, frontal operculum, parietal operculum, insular cortex	1656	4.84	−48	10	0
Left	supramarginal gyrus, postcentral gyrus, superior parietal lobule	782	5	−66	−38	36
Left	lingual gyrus, intracalcarine cortex, cuneal cortex, occipital pole, lateral occipital cortex, occipital fusiform cortex, temporal occipital fusiform cortex	702	4.24	−28	−58	4
Right	supramarginal gyrus, postcentral gyrus, superior parietal lobule	540	3.86	50	−26	56
Right	middle temporal gyrus, temporal pole, temporal fusiform cortex, pallidum	404	4.39	28	−18	−8
Right	cerebellum	278	4.05	16	−50	−22
Left	superior frontal gyrus, middle frontal gyrus, precentral gyrus	234	3.81	−24	0	62
Left	frontal pole, inferior frontal gyrus	232	4.53	−38	50	6
Right	supramarginal gyrus	151	3.7	66	−28	30
Right	precentral gyrus	109	3.94	32	−20	62
Right	superior frontal gyrus, paracingulate gyrus	100	4.28	20	16	44
Left	superior parietal lobule	94	4.03	−34	−50	60
Left	superior frontal gyrus	87	4.35	−12	−2	72
Right	precentral gyrus, posterior cingulate cortex	87	4.03	10	−24	50
Left	cerebellum	83	3.78	−16	−60	−26
Right	central opercular cortex	74	3.41	48	−10	16
Left	superior temporal gyrus	50	3.43	−56	−24	−2
Right	superior temporal gyrus	45	3.98	48	−24	−4
Right	posterior cingulate cortex	39	3.57	10	−20	24
Right	hippocampus	28	3.48	18	−36	−4
Right	inferior frontal gyrus	26	3.37	54	26	−2
Right	frontal pole	23	3.56	32	42	44
Left	middle temporal gyrus	23	3.56	−68	−48	4
Right	middle frontal gyrus	22	3.52	34	6	58
Right	posterior cingulate cortex	21	3.84	20	−24	28

The S1-I *minus* S1-R contrast was tested to show activity related specifically to processing the irrelevant S1, including possible correlates of inhibition of S1 processing in the S1-irrelevant condition. The results showed no significant S1-related activity.

**Figure 6:**
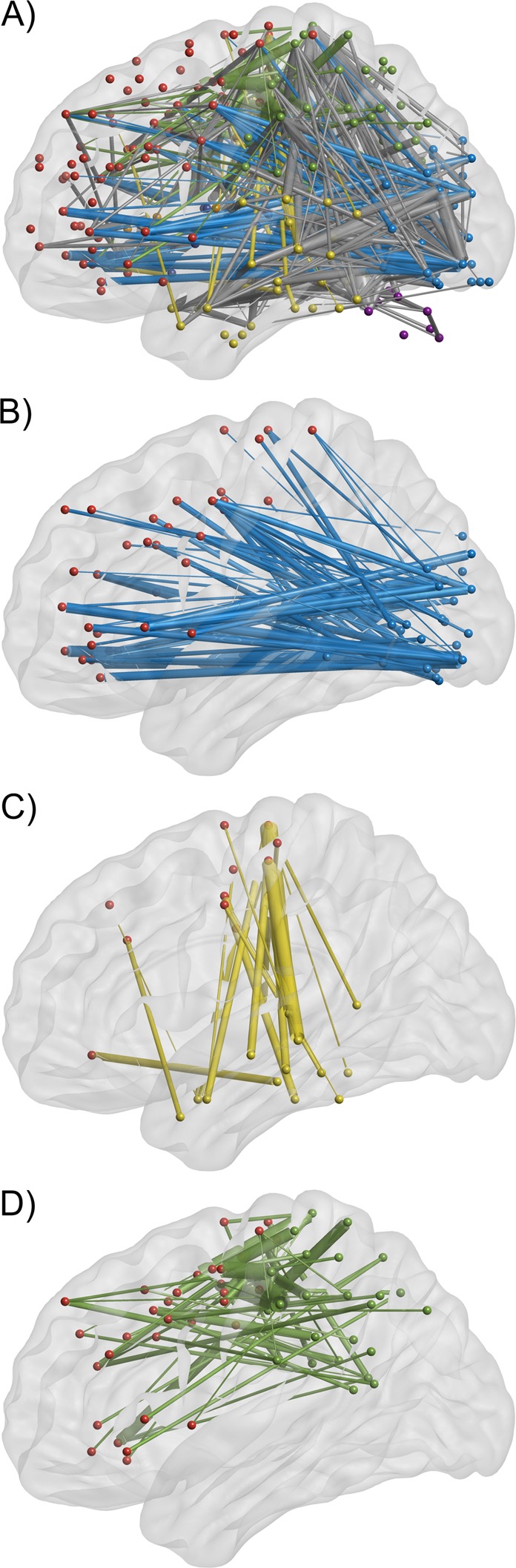
Links showing increased connectivity strength for the S1-related activity in the S1-R *minus* S1-I contrast visualized within brain space. Nodes are color-coded according to their divisional assignment: purple – cerebellum, dark blue – subcortical, light blue – occipital, green – parietal, yellow – temporal, orange – insula, red – frontal. Edge thickness reflects t-test statistic value. Note that 3D rendering of the brain does not contain cerebellum, yet nodes in the cerebellum are indicated outside the brain outline. Panel (A) shows all edges connecting posterior, i.e., occipital and temporal divisions with frontal divisions, and parietal divisions with frontal divisions. The occipito-frontal connections are colored in light blue, the temporo-frontal connections in yellow, and the parieto-frontal connections in green (in accordance to the seed division colors). Grey edges represent all other significant connections. Panel (B) shows only the occipito-frontal connections. Panel (C) shows only the temporo-frontal connections. Panel (D) shows only the parieto-frontal connections.

**Figure 7:**
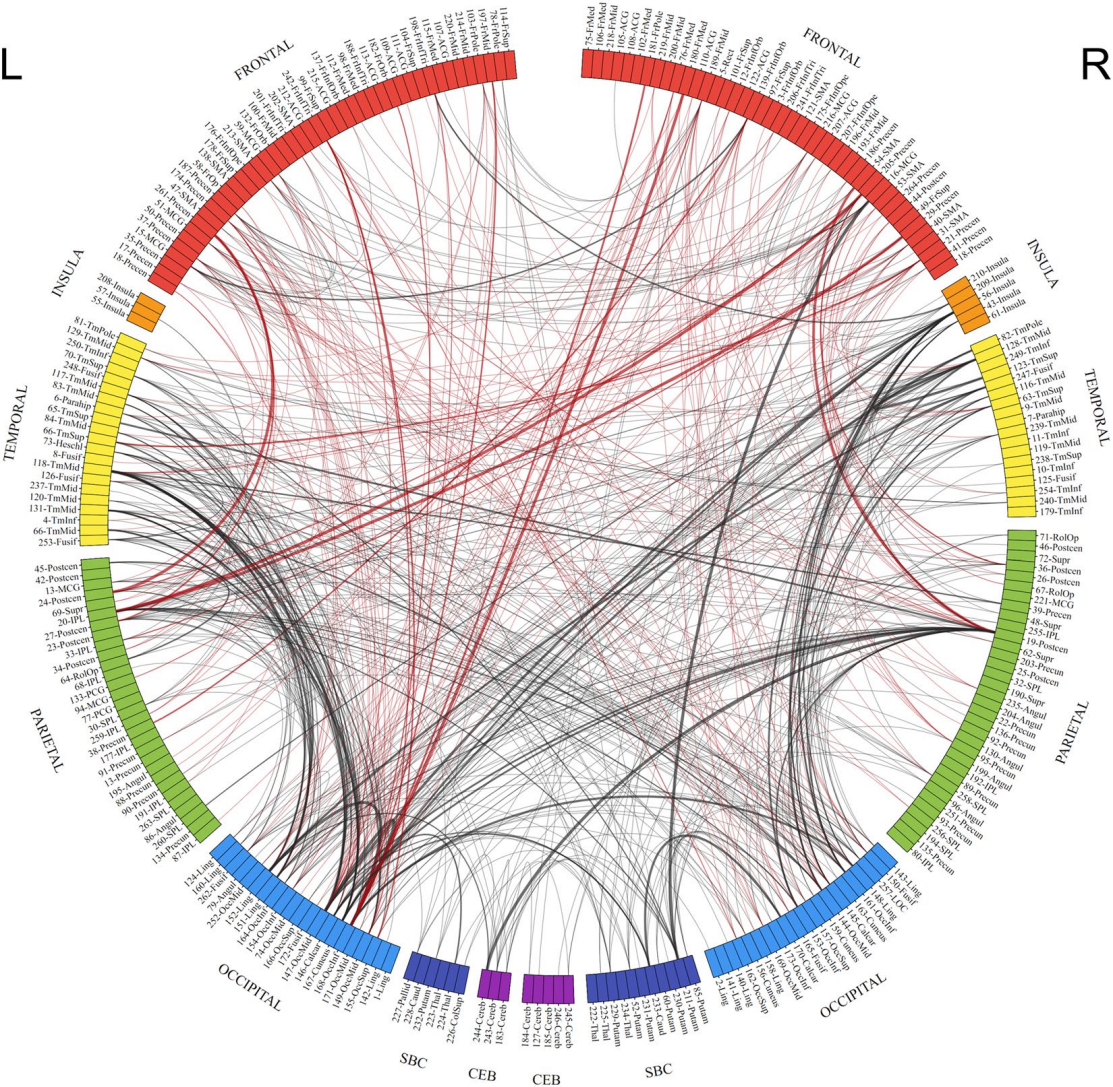
Circular representation of NBS results for the S1-related connectivity in the S1-R *minus* S1-I contrast. Nodes are color coded and assigned to anatomical divisions according to AAL labeling. Anatomical regions are color coded according to their divisional assignment: purple – cerebellum, dark blue – subcortical, light blue – occipital, green – parietal, yellow – temporal, orange – insula, red – frontal. Node abbreviation represent ROI number from Power *et al*.^62^ and anatomical label. Thickness of links connecting nodes represents *t*-values, with higher values depicted in thicker lines. Connections depicted in red represent postero-frontal connectivity (i.e. edges connecting occipital, temporal, and parietal divisions with frontal division).

**Figure 8:**
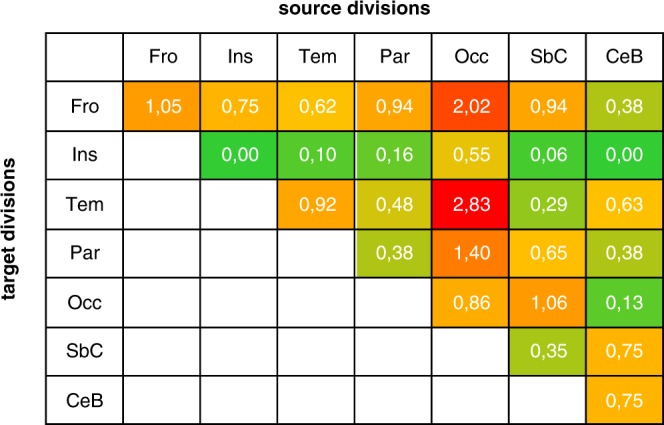
Relative density of connections between divisions, demonstrating proportions of significant connections per division. The relative density value is calculated as a ratio of significant connections from the source division to the ‘‘target division” over total number of nodes in a given “source division”. The values are sometimes bigger than 1, because one node in a source region can have more than one connection to nodes in the “target region”. Abbreviations: Fro – frontal, Ins – insula, Tem – temporal, Par – parietal, Occ – occipital, SbC – subcortical, CeB – cerebellum. Division labels are consistent with terminology used across the text. Color coding corresponds to the parameter value.

*Network based statistics (NBS)*. The NBS results for the S1-R *minus* S1-I contrast demonstrated global and widespread increase in connectivity strength, encompassing perceptual, parietal, and frontal regions of the cerebral cortex with a clearly visible pattern of increased postero-frontal connectivity (occipital, temporal, and parietal divisions connecting with frontal regions). Results of the contrast yielded a total of 688 network edges with increased connectivity, with 490 edges constituting inter-divisional connections and 198 intra-divisional connections. The overall results of the NBS analysis are shown in Fig. 6 (panel A), which depicts links between network nodes assigned to anatomical divisions. An alternative, circular representation of significant results is shown in Fig. 7, showing division affiliations of edges and postero-frontal connectivity (red lines). Figure 8 shows relative density of connections between divisions.

The largest increase of inter-division connectivity in terms of the number of connections was found for links connecting sensory regions (i.e. links between occipital and temporal areas encompassing primary and secondary visual areas) and links connecting those posterior divisions with frontal regions. We found increase of inter-division connectivity for 119 links in occipito-temporal regions, 85 links between occipital and frontal division (see Fig. 6, panel B), and 60 links between parietal and frontal regions (see Fig. 6, panel D). The posterior regions connected with nodes located on the lateral surface of frontal lobe (inferior, middle, and superior frontal gyrus), as well as at the frontal pole, frontal orbital cortex, and frontal operculum. The posterior-frontal connections also included premotor areas, as well as nodes located in primary motor areas in the precentral gyrus. Increased connectivity from posterior regions also extended to the nodes on the medial surface of the frontal lobe, mainly in the medial frontal gyrus, cingulate gyrus, and supplementary motor area. The significant temporo-frontal connectivity mainly consisted of connections between temporal regions, and motor and premotor areas in the frontal lobe (see Fig. 6, panel C).

For the intra-division connections, the largest increase of connectivity was observed within frontal areas (90 links), followed by an increase in the number of significant connections within the occipital (36 edges) and temporal divisions (36 edges). A list containing the numbers of significant connections for each anatomical division, and with postero-frontal connections depicted in red is presented in Table 5.

**Table 5:** Summary of inter- and intra-divisional links showing increased connectivity strength for S1-R *minus* S1-I contrast. Values in the brackets represent highest t-statistic for a given division pair. Numbers depicted in bold represent the occipital, temporal, and parietal to frontal connections, as shown in Fig. 6 and Fig. 7.

Anatomical Division	Frontal	Insula	Temporal	Parietal	Occipital	Subcortical	Cerebellum
Frontal	90 (4.54)	6 (4.44)	**24 (4.45)**	**60 (5.12)**	**85 (5.35)**	16 (5.19)	3 (3.66)
Insula	6 (4.44)	0 (−)	4 (4.21)	10 (4.38)	23 (4.92)	1 (3.89)	0 (−)
Temporal	**24 (4.45)**	4 (4.21)	36 (4.94)	31 (4.58)	119 (5.00)	5 (4.29)	5 (3.64)
Pariertal	**60 (5.12)**	10 (4.38)	31 (4.58)	24 (4.46)	59 (4.67)	11 (4.37)	3 (5.36)
Occipital	**85 (5.35)**	23 (4.92)	119 (5.00)	59 (4.67)	36 (5.57)	18 (4.78)	1 (4.34)
Subcortical	16 (5.19)	1 (3.89)	5 (4.29)	11 (4.37)	18 (4.78)	6 (4.07)	6 (3.81)
Cerebellum	3 (3.66)	0 (−)	5 (3.64)	3 (5.36)	1 (4.34)	6 (3.81)	6 (3.85)

The S1-I *minus* S1-R contrast was tested to show connectivity related to processing of the irrelevant S1. The results showed no significant S1-I-related increase in connectivity strength.

### Discussion of the fMRI experiment

#### Event file account

Based on the previous imaging studies on event files and sensorimotor binding^48–52^ we expected that processing of the relevant S1 will entail activation of the underlying distributed sensorimotor network including visual areas, supplementary motor, premotor, and primary motor areas, parietal and prefrontal association areas, and plausibly also the dorsolateral prefrontal executive area.

The obtained results of both the standard whole-brain mass-univariate analysis and the connectivity analyses are generally in line with these expectations. Processing of the relevant S1 was associated with activation of visual areas in the occipital and temporal cortex (cf.^50,51^), sensorimotor association areas in the posterior parietal cortex (cf.^81,82^), areas related to response preparation in the supplementary motor, premotor, and primary motor regions (cf.^48–51^), and association and executive areas in frontal and prefrontal regions (cf.^52^) presumably involved in goal-oriented processing. Note that the premotor and motor areas were activated despite the fact that the motor response had to be postponed until the go signal, which resembles findings from studies in which no overt response was required and still those areas, related to response preparation, were activated^56,83^. This pattern of activation was complemented by the connectivity results for the same S1-R minus S1-I contrast. We found a strong increase of connectivity strength between posterior and anterior cortical regions. In particular, the strength increased for the occipito-frontal and parieto-frontal connections. The connected nodes in the frontal lobes included the supplementary motor, premotor, and primary motor areas, as well as the dorsolateral prefrontal cortex. These frontal nodes were connected with visual areas in the occipital lobes, and areas in the parietal lobes, which might underlie the sensorimotor association processes.

It should also be noted here that the comparison of the main effects in the two experimental conditions revealed a large difference in terms of both a number and locations of the activated regions. This result is in line with our assumption that the relevance manipulation entails qualitative differences between the two conditions, thereby presumably also qualitatively distinct neural processes involved in both cases.

In conclusion, the present findings seem to indicate that on the brain level, the establishment and activation of S-R link entails involvement of the whole sensorimotor network, including the nodes representing S-codes, R-codes, S-R associative codes, and control mechanisms^17^, as well as strengthening the large-scale functional communication between these nodes.

#### S-R link activation hypothesis of P3

The question now is whether this sensorimotor network indeed constitutes the neural underpinnings of P3. We could give a positive answer here, if the S1 relevancy increased solely the P3 component. However, even though the positive peak in the difference waveform reflects entirely the S1-R-evoked P3 component, there is also a negative peak in this difference waveform emerging around S2. It is then possible that all the observed anterior activations in premotor, motor, and prefrontal cortex are correlates of the CNV (cf.^24^) and not of the P3, which would be in agreement with the stimulus evaluation account of P3. However, this explanation seems less plausible, given that the present EEG results, as well as the results of the several recent P3 studies conform better to the S-R link activation account^14,15,20,44,84,85^. More plausibly, the S1-R *minus* S1-I contrast in the fMRI measurement may reflect both P3- and CNV-related activity, and the amplitude increase of both components, P3 and CNV, may have contributed to the observed increase in connections projecting to frontal division. In this line, Nagai *et al*.^86^, in their study on CNV-related brain activity with simultaneous fMRI and EEG recording, have demonstrated that CNV is related to increased activity of the frontal midline structures: SMA, anterior cingulate, and thalamus. Therefore, the revealed in the S1-R minus S1-I contrast increase of connectivity to lateral frontal regions and increased activity within those lateral frontal areas might be related to P3. On the other hand, Fan *et al*.^87^ observed that CNV-related fMRI activity included SMA, anterior cingulate, and thalamus, as well as the middle frontal gyrus, superior frontal gyrus, superior parietal lobule, and basal ganglia. Further studies are therefore needed to delineate the neuronal underpinnings and functional relationship of P3 and CNV. As mentioned before (in the EEG discussion), one solution for removing these overlaps is to use the oddball manipulation and subtracting the activity evoked by rare and frequent stimuli. As CNV should be the same after rare and frequent stimuli, this rare minus frequent subtraction will yield the oddball-P3-related activity disentangled from CNV-related activity^20^.

Notwithstanding, the present results are not much different from findings of EEG-fMRI studies on the neural sources of P3. For example, Bledowski *et al*.^53^, in their combined EEG and fMRI study using a visual oddball task, found that P3 was generated in the inferior parietal lobe (IPL), posterior parietal cortex (PPC), and inferior temporal cortex. Mantini *et al*.^55^ also used a visual oddball task in their simultaneous EEG-fMRI study, and found even larger number of P3 generators, including the supplementary motor area, the anterior cingulate, the middle and superior frontal cortex, the insula, the posterior parietal cortex, and the right temporo-parietal junction. Notably, they have found within the network underlying P3 the areas that previously were not considered as related to P3 (e.g.,^53,88^), and concluded that P3 is actually associated with the response of the dorsal and ventral fronto-parietal attention networks^27^, as the P3 across-trial variability was correlated with the time-course of activations within these networks. Kiehl and colleagues^89^ analyzed a large fMRI data sample (*N* = 100) and found that processing of auditory oddball stimuli (typically evoking a large P3) was associated with 34 specified regions of interest, and six previously not observed areas, including the activated in the present study: inferior and middle frontal gyri, precentral gyrus, and cerebellum. More recently, Strobel *et al*.^56^ s reported similar fronto-parietal activations, as well as the anterior cingulate cortex, and premotor and supplementary motor areas for target processing in an auditory oddball task as the activations contributing to the target evoked P3. Thus, although the results of those studies encompass a wide spectrum of the cortical regions, the overall pattern of those wide-spread activations underlying P3, in both visual and auditory tasks, is relatively similar to the present results and suggest that the activity underlying P3 is not constrained to regions strictly related to stimulus evaluation processes.

## Conclusions

The aim of the present study was twofold. In the EEG experiment we examined whether the P3b (P3) component of electrophysiological event-related potential may be related to stimulus-response coupling, and more specifically, to activation of S-R links. In the fMRI experiment we aimed to identify the brain network possibly constituting the neuronal underpinnings of this S-R coupling, and explored whether such sensorimotor network may be the source of P3 interpreted as activation of S-R links. The obtained results showed that presenting a stimulus that provides all information about the required response increased the P3 and CNV components of ERPs, and activates the large-scale postero-anterior sensorimotor network. In line with several recent studies, the EEG results suggest that P3, rather than being unrelated to response processing, may reflect a bridging step between sensory encoding and response execution, possibly the activation of established S-R links, as proposed by Verleger *et al*.^14,15^. We define these links as a well-established (by practice) sensorimotor representation, in which sensory and motor codes are bound into a ready to use, integrated “event file”^16^. The results of the fMRI experiment suggest that the brain basis of such event files may be formed by a distributed sensorimotor network, comprised of well-connected and integrated nodes or modules in visual areas, premotor and motor areas, parietal and frontal association areas, and prefrontal executive areas, in line with previous imaging studies on the brain basis of event files. This sensorimotor network may possibly underlie the P3 component. However, further studies, using more refined experimental designs or a simultaneous fMRI-EEG acquisition, are needed to disentangle P3-related activity from overlapping CNV-related anticipatory activity, and to determine the nature of the functional relationship between P3 and CNV.

## Supplementary information

Supplementary information.
