# Supplementary material for: The response relevance of visual stimuli modulates the P3 component and the underlying sensorimotor network
Source: Sci Rep. 2020 Mar 2;10:3818. doi: 10.1038/s41598-020-60268-z (PMC7052248; doi:10.1038/s41598-020-60268-z)
Supplement: Supplementary file 1 — Supplementary information. [file 41598_2020_60268_MOESM1_ESM.pdf]

SUPPLEMENTARY INFORMATION

The response relevance of visual stimuli modulates the P3 component and the underlying sensorimotor network

Dariusz Asanowicz [a], Krzysztof Gociewicz [a],

Marcin Koculak [a], Karolina Finc [b], Kamil Bonna [b], Axel Cleeremans [c], Marek Binder [a]

[a] Institute of Psychology, Jagiellonian University, Kraków, Poland

[b] Centre for Modern Interdisciplinary Technologies, Nicolaus Copernicus University, Toruń, Poland

[c] Consciousness, Cognition, and Computation Group, Université Libre de Bruxelles, Brussels, Belgium

Table S1. Summary of each ROI's abbreviation, full anatomical label, hemisphere, MNI coordinate, and original ROI code from Power et al.[72]

Anatomical Division	Hemisphere	Abbreviation	Full Anatomical Label	MNI Coordinates			ROI Code from Power et al.
				X	Y	Z	
Frontal	Left	114_FrSup	Superior frontal gyrus	-20	57	24	114
Frontal	Left	78_FrPole	Frontal Pole	-17	57	-3	78
Frontal	Left	197_FrMid	Middle frontal gyrus	-33	49	9	197
Frontal	Left	103_FrPole	Frontal pole	-11	48	42	103
Frontal	Left	214_FrMid	Middle frontal gyrus	-27	46	25	214
Frontal	Left	220_FrMid	Middle frontal gyrus	-38	45	21	220
Frontal	Left	107_ACG	Cingulate gyrus, anterior part	-7	45	4	107
Frontal	Left	115_FrMed	Medial frontal gyrus	-8	42	27	115
Frontal	Left	198_FrInfTri	Inferior frontal gyrus, triangular part	-40	40	2	198
Frontal	Left	104_FrSup	Superior frontal gyrus	-20	39	42	104
Frontal	Left	111_ACG	Cingulate gyrus, anterior part	-11	39	12	111
Frontal	Left	109_ACG	Cingulate gyrus, anterior part	-3	39	-4	109
Frontal	Left	182_FrOrb	Frontal orbital cortex	-20	36	-15	182
Frontal	Left	113_ACG	Cingulate gyrus, anterior part	-3	36	20	113
Frontal	Left	188_FrInfTri	Inferior frontal gyrus, triangular part	-41	33	24	188
Frontal	Left	98_FrMed	Medial frontal gyrus	-11	33	54	98
Frontal	Left	112_FrMed	Medial frontal gyrus	-3	32	39	112
Frontal	Left	137_FrInfOrb	Inferior frontal gyrus, orbital part	-44	27	-9	137
Frontal	Left	215_ACG	Cingulate gyrus, anterior part	-1	25	30	215
Frontal	Left	99_FrSup	Superior frontal gyrus	-17	23	54	99
Frontal	Left	242_FrInfTri	Inferior frontal gyrus, triangular part	-47	21	2	242
Frontal	Left	212_ACG	Cingulate gyrus, anterior part	-11	21	27	212
Frontal	Left	202_SMA	Supplementary Motor Area	-4	21	46	202
Frontal	Left	201_FrInfTri	Inferior frontal gyrus, triangular part	-41	20	31	201
Frontal	Left	100_FrMid	Middle frontal gyrus	-35	15	51	100
Frontal	Left	132_FrOrb	Frontal orbital cortex	-29	15	-15	132
Frontal	Left	59_MCG	Cingulate gyrus, middle part	-6	13	36	59
Frontal	Left	213_SMA	Supplementary Motor Area	-2	10	45	213
Frontal	Left	176_FrInfOpe	Inferior frontal gyrus, opercular part	-45	7	24	176
Frontal	Left	178_FrSup	Superior frontal gyrus	-23	6	63	178
Frontal	Left	138_SMA	Supplementary Motor Area	-11	6	66	138
Frontal	Left	58_FrOp	Frontal operculum	-49	5	0	58
Frontal	Left	187_Precen	Precentral gyrus	-40	2	33	187
Frontal	Left	174_Precen	Precentral gyrus	-43	-2	45	174
Frontal	Left	47_SMA	Supplementary Motor Area	-4	-2	53	47
Frontal	Left	261_Precen	Precentral gyrus	-32	-5	53	261
Frontal	Left	51_MCG	Cingulate gyrus, middle part	-11	-6	42	51
Frontal	Left	50_Precen	Precentral gyrus	-17	-9	69	50
Frontal	Left	37_Precen	Precentral gyrus	-38	-18	66	37
Frontal	Left	15_MCG	Cingulate gyrus, middle part	-1	-18	46	15
Frontal	Left	35_Precen	Precentral gyrus	-14	-21	72	35
Frontal	Left	17_Precen	Precentral gyrus	-8	-24	63	17
Frontal	Left	18_Precen	Precentral gyrus	-8	-36	69	18

Insula	Left	208_Insula	Insula	-34	16	3	208
Insula	Left	57_Insula	Insula	-33	0	6	57
Insula	Left	55_Insula	Insula	-43	-3	10	55
Temporal	Left	81_TmPole	Temporal pole	-41	9	-30	81
Temporal	Left	129_TmMid	Middle temporal gyrus	-50	0	-24	129
Temporal	Left	250_TmInf	Inferior temporal gyrus	-47	-9	-36	250
Temporal	Left	70_TmSup	Superior temporal gyrus	-53	-12	12	70
Temporal	Left	248_Fusif	Fusiform gyrus	-29	-12	-33	248
Temporal	Left	117_TmMid	Middle temporal gyrus	-53	-15	-9	117
Temporal	Left	83_TmMid	Middle temporal gyrus	-65	-24	-15	83
Temporal	Left	6_Parahip	Parahippocampal gyrus	-20	-24	-18	6
Temporal	Left	65_TmSup	Superior temporal gyrus	-58	-27	13	65
Temporal	Left	84_TmMid	Middle temporal gyrus	-55	-27	-14	84
Temporal	Left	66_TmSup	Superior temporal gyrus	-47	-28	5	66
Temporal	Left	73_Heschl	Heschl's gyrus	-29	-29	12	73
Temporal	Left	8_Fusif	Fusiform gyrus	-35	-30	-24	8
Temporal	Left	118_TmMid	Middle temporal gyrus	-55	-31	-4	118
Temporal	Left	126_Fusif	Fusiform gyrus	-32	-39	-15	126
Temporal	Left	237_TmMid	Middle temporal gyrus	-53	-41	12	237
Temporal	Left	120_TmMid	Middle temporal gyrus	-65	-42	-6	120
Temporal	Left	131_TmMid	Middle temporal gyrus	-47	-43	0	131
Temporal	Left	4_TmInf	Inferior temporal gyrus	-53	-45	-24	4
Temporal	Left	66_TmMid	Middle temporal gyrus	-54	-51	8	236
Temporal	Left	253_Fusif	Fusiform gyrus	-44	-51	-21	253
Parietal	Left	45_Postcen	Postcentral gyrus	-51	-13	24	45
Parietal	Left	42_Postcen	Postcentral gyrus	-48	-14	34	42
Parietal	Left	13_MCG	Cingulate gyrus, middle part	-14	-21	39	14
Parietal	Left	24_Postcen	Postcentral gyrus	-39	-22	52	24
Parietal	Left	69_Supr	Supramarginal gyrus	-51	-24	22	69
Parietal	Left	20_IPL	Inferior parietal lobule	-52	-25	41	20
Parietal	Left	27_Postcen	Postcentral gyrus	-38	-30	66	27
Parietal	Left	23_Postcen	Postcentral gyrus	-23	-33	69	23
Parietal	Left	33_IPL	Inferior parietal lobule	-44	-34	44	33
Parietal	Left	34_Postcen	Postcentral gyrus	-21	-34	58	34
Parietal	Left	64_RolOp	Rolandic operculum	-37	-35	16	64
Parietal	Left	68_IPL	Inferior parietal lobule	-48	-36	24	68
Parietal	Left	133_PCG	Cingulate gyrus, posterior part	-3	-37	30	133
Parietal	Left	94_MCG	Cingulate gyrus, middle part	-3	-39	42	94
Parietal	Left	77_PCG	Cingulate gyrus, posterior part	-12	-41	1	77
Parietal	Left	30_SPL	Superior parietal lobule	-29	-45	57	30
Parietal	Left	259_IPL	Inferior parietal lobule	-32	-48	44	259
Parietal	Left	38_Precun	Precuneus	-17	-48	69	38
Parietal	Left	177_IPL	Inferior parietal lobule	-51	-50	39	177
Parietal	Left	91_Precun	Precuneus	-3	-50	12	91
Parietal	Left	13_Precun	Precuneus	-8	-54	57	13
Parietal	Left	195_Angul	Angular gyrus	-41	-56	41	195
Parietal	Left	88_Precun	Precuneus	-7	-56	25	88
Parietal	Left	90_Precun	Precuneus	-11	-57	14	90

Parietal	Left	191_IPL	Inferior parietal lobule	-28	-59	44	191
Parietal	Left	263_SPL	Superior parietal lobule	-17	-60	60	263
Parietal	Left	86_Angul	Angular gyrus	-43	-65	31	86
Parietal	Left	260_SPL	Superior parietal lobule	-26	-71	33	260
Parietal	Left	134_Precun	Precuneus	-7	-72	38	134
Parietal	Left	87_IPL	Inferior parietal lobule	-38	-75	39	87
Occipital	Left	124_Ling	Lingual gyrus	-25	-41	-8	124
Occipital	Left	160_Ling	Lingual gyrus	-15	-53	-2	160
Occipital	Left	262_Fusif	Fusiform gyrus	-40	-60	-10	262
Occipital	Left	79_Angul	Angular gyrus	-44	-61	18	79
Occipital	Left	252_OccMid	Middle occipital gyrus	-50	-63	3	252
Occipital	Left	152_Ling	Lingual gyrus	-17	-68	3	152
Occipital	Left	151_Ling	Lingual gyrus	-14	-72	-9	151
Occipital	Left	164_OccInf	Inferior occipital gyrus	-40	-73	-2	164
Occipital	Left	154_OccInf	Inferior occipital gyrus	-44	-75	-12	154
Occipital	Left	74_OccMid	Middle occipital gyrus	-39	-75	22	74
Occipital	Left	166_OccSup	Superior occipital gyrus	-16	-77	30	166
Occipital	Left	172_Fusif	Fusiform gyrus	-31	-78	-15	172
Occipital	Left	147_OccMid	Middle occipital gyrus	-27	-79	16	147
Occipital	Left	146_Calcar	Calcarine gyrus	-8	-80	5	146
Occipital	Left	167_Cuneus	Cuneus	-3	-81	18	167
Occipital	Left	168_OccInf	Inferior occipital gyrus	-38	-87	-9	168
Occipital	Left	171_OccMid	Middle occipital gyrus	-25	-89	0	171
Occipital	Left	149_OccMid	Middle occipital gyrus	-23	-90	15	149
Occipital	Left	155_OccSup	Superior occipital gyrus	-14	-90	27	155
Occipital	Left	142_Ling	Lingual gyrus	-11	-93	-15	142
Occipital	Left	1_Ling	Lingual gyrus	-23	-96	-15	1
Subcortical	Left	227_Pallid	Pallidum	-21	4	-2	227
Subcortical	Left	228_Caud	Caudate	-15	0	10	228
Subcortical	Left	232_Putam	Putamen	-30	-14	1	232
Subcortical	Left	223_Thal	Thalamus	-2	-16	13	223
Subcortical	Left	224_Thal	Thalamus	-10	-21	8	224
Subcortical	Left	226_ColSup	Superior Colliculus	-5	-30	-3	226
Cerebellum	Left	244_Cereb	Cerebellum	-30	-55	-25	244
Cerebellum	Left	243_Cereb	Cerebellum	-15	-65	-20	243
Cerebellum	Left	183_Cereb	Cerebellum	-16	-75	-25	183
Frontal	Right	75_FrMed	Medial frontal gyrus	5	60	3	75
Frontal	Right	106_FrMed	Medial frontal gyrus	5	57	27	106
Frontal	Right	218_FrMid	Middle frontal gyrus	29	49	20	218
Frontal	Right	105_ACG	Cingulate gyrus, anterior part	5	48	21	105
Frontal	Right	108_ACG	Cingulate gyurs, anterior part	8	48	9	108
Frontal	Right	102_FrMed	Medial frontal gyrus	11	48	42	102
Frontal	Right	181_FrPole	Frontal Pole	32	48	-6	181
Frontal	Right	219_FrMid	Middle frontal gyurs	24	43	31	219
Frontal	Right	200_FrMid	Middle frontal gyrus	41	43	4	200
Frontal	Right	76_FrMed	Medial frontal gyrus	8	42	-9	76
Frontal	Right	180_FrMed	Medial frontal gyrus	23	39	-9	180
Frontal	Right	110_ACG	Cingulate gyrus, anterior part	7	37	0	110

Frontal	Right	189_FrMid	Middle frontal gyrus	36	37	20	189
Frontal	Right	5_Rect	Gyrus rectus	8	36	-18	5
Frontal	Right	101_FrSup	Superior frontal gyrus	20	33	42	101
Frontal	Right	12_FrInfOrb	Inferior frontal gyrus, orbital part	32	33	-6	12
Frontal	Right	122_ACG	Cingulate gyrus, anterior part	11	30	24	122
Frontal	Right	139_FrInfOrb	Inferior frontal gyrus, orbital part	47	30	-6	139
Frontal	Right	97_FrSup	Superior frontal gyrus	21	27	50	97
Frontal	Right	3_FrInfOrb	Inferior frontal gyrus, orbital part	23	27	-12	3
Frontal	Right	206_FrInfTri	Inferior frontal gyrus, triangular part	29	27	30	206
Frontal	Right	241_FrInfTri	Inferior frontal gyrus, triangular part	50	27	6	241
Frontal	Right	121_SMA	Supplementary Motor Area	11	24	60	121
Frontal	Right	175_FrInfOpe	Inferior frontal gyrus, opercular part	45	19	30	175
Frontal	Right	216_MCG	Cingulate gyrus, middle part	4	18	39	216
Frontal	Right	207_ACG	Cingulate gyurs, anterior part	9	17	30	217
Frontal	Right	207_FrInfOpe	Inferior frontal gyrus, opercular part	45	17	14	207
Frontal	Right	196_FrMid	Middle frontal gyrus	37	13	42	196
Frontal	Right	193_FrMid	Middle frontal gyrus	29	9	57	193
Frontal	Right	186_Precen	Precentral gyrus	44	5	35	186
Frontal	Right	54_SMA	Supplementary Motor Area	5	3	51	54
Frontal	Right	205_Precen	Precentral gyrus	39	-5	48	205
Frontal	Right	16_MCG	Cingulate gyrus, middle part	8	-6	45	16
Frontal	Right	53_SMA	Supplementary Motor Area	11	-6	69	53
Frontal	Right	264_Precen	Precentral gyrus	26	-9	54	264
Frontal	Right	44_Precen	Precentral gyrus	48	-10	34	44
Frontal	Right	49_FrSup	Frontal superior gyrus	17	-12	63	49
Frontal	Right	29_Precen	Precentral gyrus	41	-12	57	29
Frontal	Right	40_SMA	Supplementary Motor Area	2	-21	57	40
Frontal	Right	31_SMA	Supplementary Motor Area	8	-21	72	31
Frontal	Right	21_Precen	Precentral gyrus	26	-21	69	21
Frontal	Right	41_Precen	Precentral gyrus	35	-21	45	41
Frontal	Right	18_Precen	Precentral gyrus	18	-32	58	28
Insula	Right	210_Insula	Insula	35	27	3	210
Insula	Right	209_Insula	Insula	34	17	7	209
Insula	Right	56_Insula	Insula	47	4	3	56
Insula	Right	43_Insula	Insula	34	-13	16	43
Insula	Right	61_Insula	Insula	30	-29	14	61
Temporal	Right	82_TmPole	Temporal pole	44	12	-24	82
Temporal	Right	128_TmMid	Middle temporal gyrus	50	3	-24	128
Temporal	Right	249_TmInf	Inferior temporal gyrus	47	-6	-33	249
Temporal	Right	123_TmSup	Superior temporal gyrus	50	-6	-12	123
Temporal	Right	247_Fusif	Fusiform gyrus	32	-15	-30	247
Temporal	Right	116_TmMid	Middle temporal gyrus	62	-15	-15	116
Temporal	Right	63_TmSup	Superior temporal gyrus	55	-19	10	63
Temporal	Right	9_TmMid	Middle temporal gyrus	62	-27	-15	9
Temporal	Right	7_Parahip	Parahippocampal gyrus	17	-30	-15	7
Temporal	Right	239_TmMid	Middle temporal gyrus	49	-31	-2	239
Temporal	Right	11_TmInf	Inferior temporal gyrus	53	-33	-14	11
Temporal	Right	119_TmMid	Middle temporal gyrus	62	-33	-6	119

Temporal	Right	238_TmSup	Superior temporal gyrus	49	-35	9	238
Temporal	Right	10_TmInf	Inferior temporal gyrus	50	-36	-24	10
Temporal	Right	125_Fusif	Fusiform gyrus	26	-39	-11	125
Temporal	Right	254_TmInf	Inferior temporal gyrus	44	-48	-15	254
Temporal	Right	240_TmMid	Middle temporal gyrus	53	-48	12	240
Temporal	Right	179_TmInf	Inferior temporal gyrus	56	-54	-12	179
Parietal	Right	71_RolOp	Rolandic operculum	53	-9	16	71
Parietal	Right	46_Postcen	Postcentral gyrus	62	-12	27	46
Parietal	Right	72_Supr	Supramarginal gyrus	56	-21	30	72
Parietal	Right	36_Postcen	Postcentral gyrus	39	-24	54	36
Parietal	Right	26_Postcen	Postcentral gyrus	47	-24	42	26
Parietal	Right	67_RolOp	Rolandic operculum	41	-26	21	67
Parietal	Right	221_MCG	Cingulate gyrus, middle part	1	-27	30	221
Parietal	Right	39_Precen	Precentral gyrus	1	-31	58	39
Parietal	Right	48_Supr	Supramarginal gyrus	51	-31	34	48
Parietal	Right	255_IPL	Inferior parietal lobule	44	-33	48	255
Parietal	Right	19_Postcen	Postcentral gyrus	11	-36	72	19
Parietal	Right	62_Supr	Supramarginal gyrus	62	-36	21	62
Parietal	Right	203_Precun	Precuneus	9	-41	48	203
Parietal	Right	25_Postcen	Postcentral gyrus	26	-42	57	25
Parietal	Right	32_SPL	Superior parietal lobule	20	-45	66	32
Parietal	Right	190_Supr	Supramarginal gyrus	46	-45	44	190
Parietal	Right	235_Angul	Angular gyrus	51	-45	22	235
Parietal	Right	204_Angul	Angular gyrus	52	-47	36	204
Parietal	Right	22_Precun	Precuneus	8	-48	69	22
Parietal	Right	136_Precun	Precuneus	3	-50	48	136
Parietal	Right	92_Precun	Precuneus	7	-50	29	92
Parietal	Right	130_Angul	Angular gyrus	44	-52	28	130
Parietal	Right	95_Precun	Precuneus	10	-55	16	95
Parietal	Right	199_Angul	Angular gyrus	31	-55	42	199
Parietal	Right	192_IPL	Inferior parietal lobule	41	-55	45	192
Parietal	Right	89_Precun	Precuneus	5	-60	33	89
Parietal	Right	258_SPL	Superior parietal lobule	23	-60	57	258
Parietal	Right	96_Angul	Angular gyrus	49	-61	34	96
Parietal	Right	251_Precun	Precuneus	8	-63	57	251
Parietal	Right	93_Precun	Precuneus	14	-64	24	93
Parietal	Right	256_SPL	Superior parietal lobule	20	-66	45	256
Parietal	Right	194_SPL	Superior parietal lobule	35	-66	38	194
Parietal	Right	135_Precun	Precuneus	10	-67	39	135
Parietal	Right	80_IPL	Inferior parietal lobule	41	-73	26	80
Occipital	Right	143_Ling	Lingual gyrus	17	-48	-9	143
Occipital	Right	150_Fusif	Fusiform gyrus	26	-60	-9	150
Occipital	Right	257_LOC	Lateral occipital cortex	44	-60	4	257
Occipital	Right	148_Ling	Lingual gyrus	19	-66	1	148
Occipital	Right	161_OccInf	Inferior occipital gyrus	40	-66	-8	161
Occipital	Right	163_Cuneus	Cuneus	5	-72	21	163
Occipital	Right	145_Calcar	Calcarine gyrus	8	-72	9	145
Occipital	Right	144_OccMid	Middle occipital gyrus	38	-73	13	144

Occipital	Right	159_Cuneus	Cuneus	14	-77	28	159
Occipital	Right	157_OccSup	Superior occipital gyrus	27	-77	23	157
Occipital	Right	153_OccInf	Inferior occipital gyrus	41	-78	-12	153
Occipital	Right	165_Fusif	Fusiform gyrus	25	-79	-16	165
Occipital	Right	170_Calcar	Calcarine gyrus	6	-81	4	170
Occipital	Right	173_OccInf	Inferior occipital gyrus	35	-81	0	173
Occipital	Right	169_OccMid	Middle occipital gyrus	35	-84	11	169
Occipital	Right	158_Ling	Lingual gyrus	19	-85	-4	158
Occipital	Right	156_Cuneus	Cuneus	14	-87	33	156
Occipital	Right	162_OccSup	Superior occipital gyrus	23	-87	21	162
Occipital	Right	140_Ling	Lingual gyrus	8	-90	-9	140
Occipital	Right	141_Ling	Lingual gyrus	17	-90	-15	141
Occipital	Right	2_Ling	Lingual gyrus	26	-96	-15	2
Subcortical	Right	85_Putam	Putamen	26	12	-12	85
Subcortical	Right	211_Putam	Putamen	32	12	-3	211
Subcortical	Right	230_Putam	Putamen	22	6	5	230
Subcortical	Right	60_Putam	Putamen	34	6	5	60
Subcortical	Right	233_Caud	Caudate	14	1	10	233
Subcortical	Right	231_Putam	Putamen	27	-3	7	231
Subcortical	Right	52_Putam	Putamen	35	-3	0	52
Subcortical	Right	234_Thal	Thalamus	8	-7	8	234
Subcortical	Right	229_Putam	Putamen	29	-17	4	229
Subcortical	Right	225_Thal	Thalamus	11	-20	9	225
Subcortical	Right	222_Thal	Thalamus	6	-26	1	222
Cerebellum	Right	245_Cereb	Cerebellum	22	-58	-22	245
Cerebellum	Right	246_Cereb	Cerebellum	1	-62	-18	246
Cerebellum	Right	185_Cereb	Cerebellum	34	-67	-33	185
Cerebellum	Right	127_Cereb	Cerebellum	28	-76	-31	127
Cerebellum	Right	184_Cereb	Cerebellum	17	-79	-34	184
